# The mitogen-activated protein kinase module CcSte11-CcSte7-CcPmk1 regulates pathogenicity via the transcription factor CcSte12 in *Cytospora chrysosperma*

**DOI:** 10.1007/s44154-023-00142-w

**Published:** 2024-01-16

**Authors:** Lu Yu, Yuchen Yang, Xiaolin Qiu, Dianguang Xiong, Chengming Tian

**Affiliations:** 1 State Key Laboratory of Efficient Production of Forest Resources, Beijing, China; 2 Beijing Key Laboratory for Forest Pest Control, Beijing Forestry University, Beijing, 100083 China

**Keywords:** *Cytospora chrysosperma*, MAPK, Transcription factor Ste12, Pathogenicity, Transcriptional analysis

## Abstract

The pathogen *Cytospora chrysosperma* is the causal agent of poplar canker disease and causes considerable economic losses in China. Mitogen-activated protein kinase (MAPK) cascades play a crucial role in mediating cellular responses and Pmk1-MAPKs are indispensable for pathogenic related processes in plant pathogenic fungi. In previous studies, we demonstrated that the *CcPmk1* acts as a core regulator of fungal pathogenicity by modulating a small number of master downstream targets, such as *CcSte12*. In this study, we identified and characterized two upstream components of CcPmk1: MAPKKK *CcSte11* and MAPKK *CcSte7*. Deletion of *CcSte11* and *CcSte7*, resulted in slowed growth, loss of sporulation and virulence, similar to the defects observed in the *CcPmk1* deletion mutant. In addition, CcSte11, CcSte7 and CcPmk1 interact with each other, and the upstream adaptor protein CcSte50 interact with CcSte11 and CcSte7. Moreover, we explored the global regulation network of *CcSte12* by transcriptional analysis between *CcSte12* deletion mutants and wild-type during the simulated infection process. Two hydrolase activity GO terms (GO:0004553 and GO:0016798) and starch and sucrose metabolism (mgr00500) KEGG pathway were significantly enriched in the down-regulated genes of *CcSte12* deletion mutants. In addition, a subset of glycosyl hydrolase genes and putative effector genes were significantly down-regulated in the *CcSte12* deletion mutant, which might be important for fungal pathogenicity. Especially, *CcSte12* bound to the *CcSp84* promoter region containing the TGAAACA motif. Moreover, comparison of *CcSte12*-regulated genes with *CcPmk1*-regulated genes revealed 116 overlapping regulated genes in both *CcSte12* and *CcPmk1*, including some virulence-associated genes. Taken together, the protein complexes CcSte11-CcSte7-CcPmk1 receive signals transmitted by upstream CcSte50 and transmit signals to downstream CcSte12, which regulates hydrolase, effectors and other genes to promote virulence. Overall, these results indicate that the CcPmk1-MAPK signaling pathway of *C. chrysosperma* plays a key role in the pathogenicity.

**Supplementary Information:**

The online version contains supplementary material available at 10.1007/s44154-023-00142-w.

## Introduction

Mitogen-activated protein kinase (MAPK) pathways are highly conserved signaling cascades in eukaryotes that are critical for the regulation of various biological processes (Hamel et al. 2012). In a general MAPK pathway, stimulus detection at a receptor leads to the activation of three protein kinases: MAP kinase kinase kinase (MAPKKK or MAP3K), MAP kinase kinase (MAPKK or MAP2K), and MAP kinase (MAPK) to transmit a variety of extracellular stimuli to downstream targets (Turra et al. 2014). MAP3K phosphorylate downstream MAP2K at the serine (S) or threonine (T) residues of the [S/T]-X5-[S/T] motif, the activated MAPKKs phosphorylate MAPKs at the TXY motif, and the activated MPKs phosphorylate specific downstream substrates at the SP/TP site, leading to the triggering of cellular responses (Hamel et al. 2012; Zhang et al. 2021b). In addition, MAP3K and MAP2K also include conserved Asp and Lys residues within the active site (D[L/I/V]K motif) (Hamel et al. 2012). The budding yeast *Saccharomyces cerevisiae* has five MAPK pathways, Fus3, Kss1, Slt2, Hog1 and Smk1, that regulate mating, invasive growth, cell wall integrity, osmoregulation, and ascospore formation respectively. In contrast, most filamentous fungal pathogens possess only three MAPK pathways, orthologs of the Fus3/Kss1, Slt2 and Hog1 pathways (Jiang et al. 2018).

The Fus3/Kss1 pathway is denominated as pathogenic MAPKs (Pmk1) in plant pathogenic fungi, which controls infection-related morphogenesis and invasive growth (Turra et al. 2014). The canonical Pmk1 pathway contains MAP3K Ste11, MAP2K Ste7 and MAPK Pmk1, as well as the upstream Ste50 adaptor protein and downstream transcription factors (Zhao et al. 2007; Zhang et al. 2021b). The core components of the Pmk1 pathway are well conserved in fungi, and their biological roles in pathogenic related processes have been reported in several filamentous pathogenic fungi. For example, Mst50 (ste50), Mst11 (Ste11), Mst7 (Ste7) and Pmk1 of *Magnaporthe oryzae* regulate appressorium formation and plant infection (Xu and Hamer 1996; Zhao et al. 2005; Park et al. 2006). Mst50 directly interacts with both Mst11 and Mst7, while Mst11 weakly interacts with Mst7, and the interaction between Mst7 and Pmk1 only during appressorium formation (Zhao et al. 2005; Zhao and Xu 2007). The SAM domain is essential for the activity of Mst11 and a highly conserved MAPK-docking site in Mst7 is essential for Pmk1 activation (Zhao et al. 2005, Zhao and Xu 2007). In addition, systematic characterization of *Fusarium graminearum* revealed that FgSte50-Ste11-Ste7-Pmk1 regulates fungal development and virulence (Gu et al. 2015a; Ren et al. 2019). Moreover, the pheromone module SteC (Ste11)-MkkB (Ste7)-MpkB (Pmk1)-SteD (Ste50) regulates development, stress responses and secondary metabolism in *Aspergillus fumigatus* (Dean Frawley et al. 2020).

The transcription factor Ste12, is downstream of the Fus3/Kss1 in *S. cerevisiae* (Madhani and Fink 1997; Madhani and Styles 1997). Activation by mating pheromones occurs only in cells containing a Ste12/Dig1/Dig2 complex and the activation of genes involved in filamentation depends on the Tec1/Ste12/Dig1 complex (Chou et al. 2006). Intriguingly, only the homeodomain was found at the N terminal region of Ste12 in yeast, while there were two tandem C_2_H_2_ zinc finger domains at the C-terminal region in addition to the N terminal homeodomain in many phytopathogenic fungi. Ste12 orthologues are required for pathogenicity in plenty of phytopathogenic fungi, while their pathogenic strategies are various (Rispail and Di Pietro 2009; Gu et al. 2015b; Schamber et al. 2010; Wong Sak Hoi and Dumas 2010; Tsuji et al. 2003). In *M. oryzae*, *Mst12* (Ste12) mutants cannot cause disease and showed a defect in microtubule reorganization during penetration peg formation, which possibly leads to penetration defects, in spite of forming appressoria normally (Park et al. 2002; Park et al. 2004). Furthermore, Mst12 is directly phosphorylated by Pmk1 and regulates genes associated with plant tissue colonization, including a subset of effectors involved in suppression of host immunity (Oses-Ruiz et al. 2021). By comparison, *FgSte12* is required for *F. graminearum* penetration of the cellophane sheet and host plant tissue. The defects in penetration may partially result from the low levels of secretion of the cell wall-degrading enzymes in *FgSte12* mutants (Gu et al. 2015b). Similarly, *FoSte12* of *F. oxysporum* regulates the activities of extracellular amylase and cellulase (Asunción García-Sánchez et al. 2010). Additionally, the exon skipping events were described in Ste12 ortholog in *Botrytis cinerea*, and the spliced ste12 transcripts have the defect in sclerotium formation (Schamber et al. 2010).

Although there are many researches about the Pmk1 pathway and the downstream Ste12 transcription factor in a variety of fungal pathogens, the biological functions of each Pmk1 pathway component may vary considerably among different species. And Pmk1-MAPKs play a critical role in fungal pathogenicity by various strategies. Particularly, it is worth noting that the host plants of most well-studied phytopathogenic fungi are crops. *Cytospora chrysosperma*, the causal agent of poplar canker disease, results in significant economic and ecological losses in China every year (Fan et al. 2020). *C. chrysosperma* infects woody plants through micro-wounds and various symptoms are observed on different host species and at different stages of disease development (Biggs et al. 1983; Fan et al. 2020; Kepley et al. 2015). Despite the high economic impact of poplar canker disease, efficient strategies for poplar canker disease management have not been well established.

With the purpose of developing novel approaches for poplar canker disease control in the future, we are looking forward to revealing the molecular mechanism of *C. chrysosperma* infection and pathogenesis. In our previous studies, CcPmk1, the Fus3/Kss1/Pmk1 homologous in *C. chrysosperma*, has been well characterized. *CcPmk1* is a core regulator of fungal pathogenicity and can potentially be designed as a target for broad-spectrum disease control (Xiong et al. 2021; Yu et al. 2019). *CcPmk1* can modulate and phosphorylate a series of pathogenicity-related downstream targets including secondary metabolism related genes, transcription factors and putative effector genes to promote virulence (Xiong et al. 2021; Yu et al. 2022). Especially, CcPmk1 could phosphorylate and interact with the downstream homeobox transcription factor CcSte12, which is required for fungal pathogenicity (Yu et al. 2022). However, the functions of the MAP3K Ste11 and the MAP2K Ste7 are still unclear in *C. chrysosperma*. Besides, the global regulation network of *CcSte12* remains a mystery.

The aim of the present study was to systematically dissect the roles of the CcPmk1-pathway in *C. chrysosperma*. We identified and characterized the functions of *CcSte11* and *CcSte7* in *C. chrysosperma*. *CcSte11* and *CcSte7* regulates fungal growth, development and pathogenicity, which are highly consistent with *CcPmk1*. Moreover, CcSte11, CcSte7 and CcPmk1 interact with each other, and the upstream protein CcSte50 can interact with CcSte7 and CcSte11. Furthermore, we conducted a comparative transcriptome analysis between wild type and CcSte12 deletion mutants and found *CcSte12* may regulate hydrolase genes to prompt virulence. Overall, these results suggests that the CcPmk1 pathway module is a highly conserved signaling pathway that is critical for the regulation of development and virulence in *C. chrysosperma*. Our findings lay foundation for further exploration of the pathogenesis of *C. chrysosperma*.

## Results

### Characterization and deletion of *CcSte11*, and *CcSte7* orthologues in *C. chrysosperma*

The orthologs of the MAP3K Ste11 and MAP2K Ste7 were identified in *C. chrysosperma* by the BlastP algorithm searching the genome sequence with the protein sequence of Mst11 (MGG_14847.6) and Mst7 (MGG_00800.6) from *M. oryzae*. Two homologues, *GME6160_g* and *GME2089_g*, were acquired and manually confirmed, named *CcSte11* and *CcSte7* respectively. The *CcSte11* has 2792 bp nucleotides that is interrupted by one intron and encodes a 911 amino acid (aa) protein. The *CcSte7* is 1791 bp in length with three introns, and encodes a 539 aa polypeptide. Both of them contain a protein kinase domain (IPR000719) and CcSte11 contains a sterile alpha motif (SAM) domain (IPR001660) and a Ras association (RA) domain (IPR029458) (Fig. S1A). In addition, CcSte11and CcSte7 include conserved Asp and Lys residues within the active site DIK motif (Fig. S1B).

Alignment of the predicted amino acid sequences revealed a striking high level of similarity among Ste11 and Ste7 homologs from other fungi, especially the homologs from Ascomycota (over 60%) (Fig. S1B and Tab. S1). For example, CcSte11 shared 88% identity with *Cryphonectria parasitica* CpSte11, 76% identity with *M. oryzae* Mst11, 76% identity with *Colletotrichum gloeosporioides* CgSte11. In addition, CcSte7 shared 86% identity with *M. oryzae* Mst7, 84% identity with *C. parasitica* CpSte7, 74% identity with *C. gloeosporioides* CgSte7. Furthermore, phylogenetic analysis indicated that the fungal Pmk1-MAPK members could be categorized into three separate clades including the Ste11 group, Ste7 group, and Pmk1 group (Fig. S2).

To investigate the functional roles of *CcSte11* and *CcSte7* in *C. chrysosperma*, we deleted *CcSte11* and *CcSte7* respectively via homology-dependent targeted gene replacement. The deletion mutants were verified by PCR and Southern blot and we generated two *CcSte11* and two *CcSte7* deletion mutants (Fig. S3). The Δ*CcSte11–4* and Δ*CcSte7–5* mutant strains were used for the subsequent phenotypic analysis, and are hereafter referred to as Δ*CcSte11* and Δ*CcSte7*. The complemented strain Δ*CcSte11/STE11* and Δ*CcSte7/STE7*, generated by transforming genomic copies of *CcSte11* and *CcSte7* into protoplasts of Δ*CcSte11* and Δ*CcSte7* (Fig. S3).

### CcPmk1-MAPKs are critical for fungal development and virulence

**Fig. 1:**
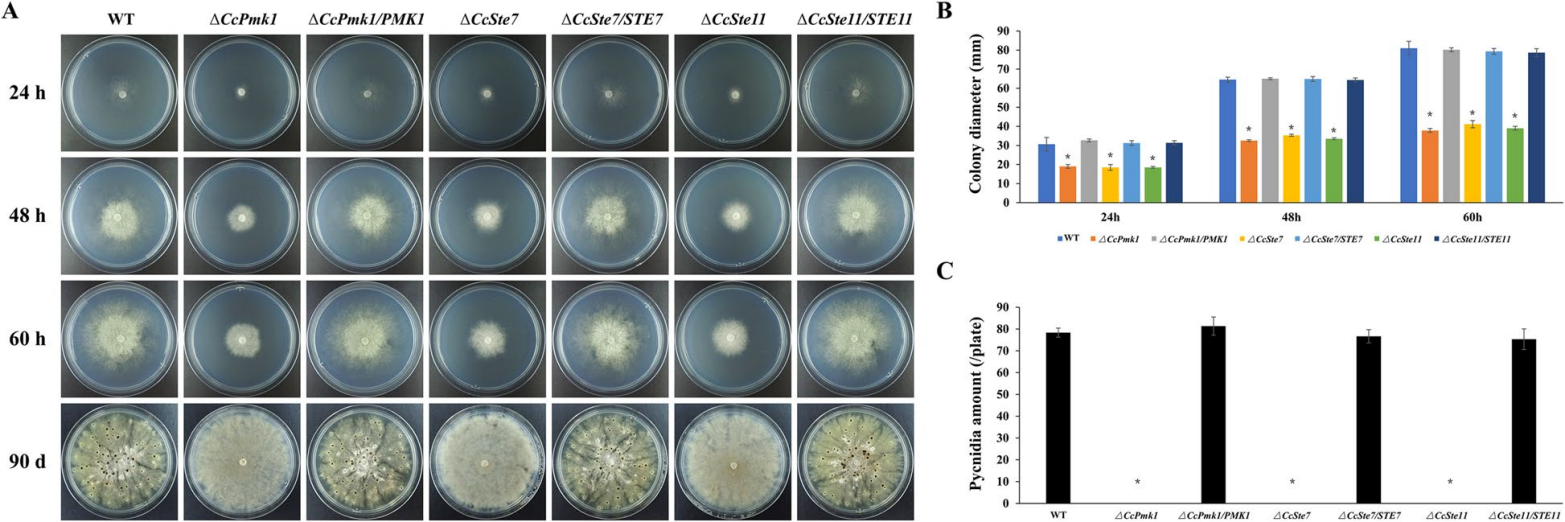
CcSte11, CcSte7 and CcPmk1 are required for fungal development. **A** Colony morphologies of the wild-type, mutants, and complemented strains after 24 h, 48 h, 60 h and 90 d of growth on PDA plates. **B** Colony diameters of these strains on PDA plates. **C** Quantification of pycnidia production per plate in these strains. The asterisks indicate significant differences (*P* < 0.05)

To determine whether the CcPmk1-MAPKs contribute to regulating vegetative growth in *C. chrysosperma*, the colony diameters of each strain were measured. It was observed that each mutant exhibited a significantly smaller colony diameter in comparison to the wild-type strain, with the similarly degree of reduction (Fig. 1). The conidiation ability of the Δ*CcSte11* and Δ*CcSte7* mutants was completely abolished, which were also found in Δ*CcPmk1* (Fig. 1). Complementation of each gene restored the wild type phenotype.

**Fig. 2:**
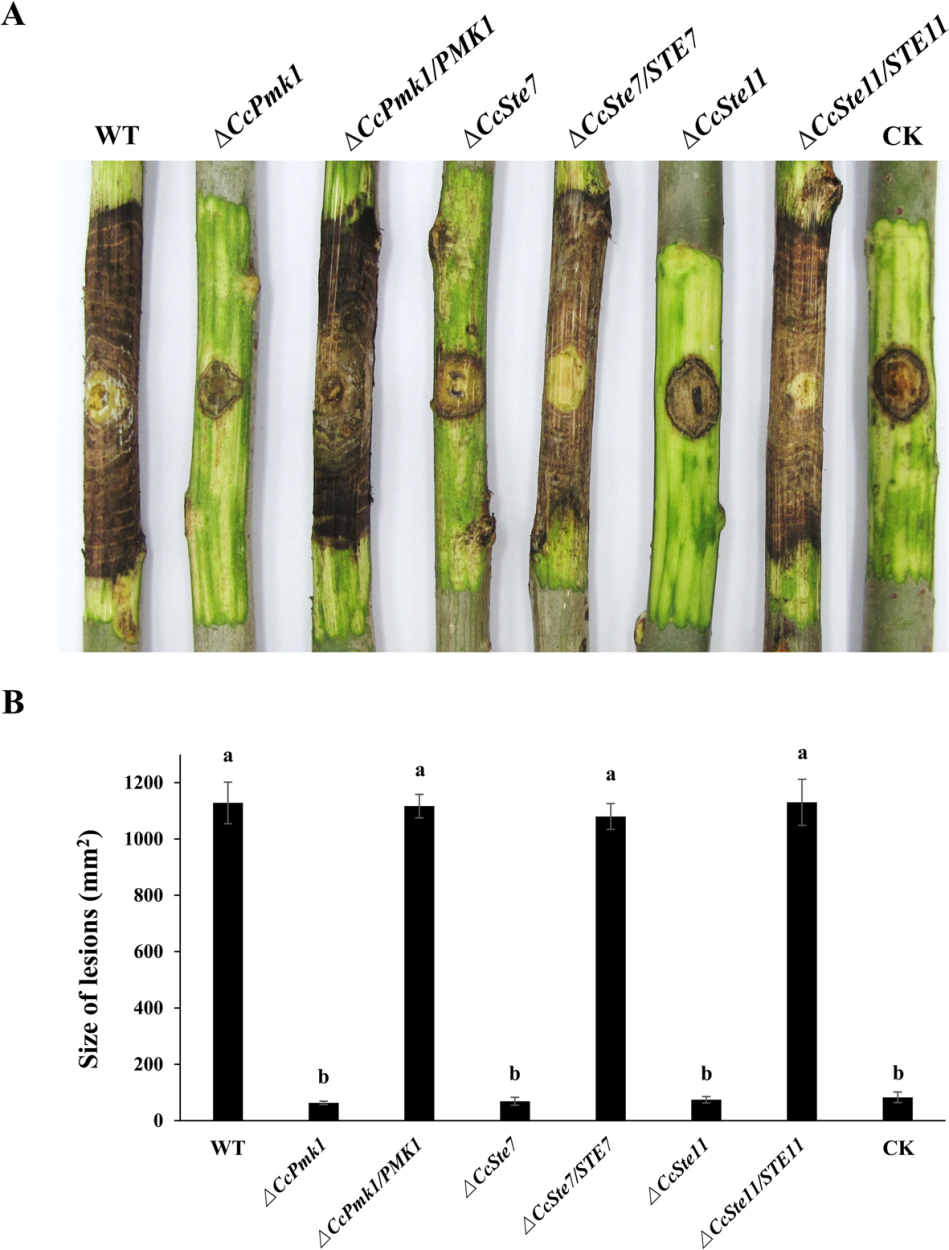
The *C. chrysosperma* CcPmk1-MAPKs regulate the fungal virulence. **A** Infection symptoms on detached poplar twigs inoculated with the wild-type, mutants, and complemented strains for 15 days. CK indicates twigs inoculated with 5 mm PDA agar plugs. **B** The bar chart exhibits area of lesions produced by the different strains on twigs. The different letters indicate significant differences (*P* < 0.05)

Then, we investigated the pathogenicity of Δ*CcSte11* and Δ*CcSte7* using detached poplar twigs. The lesions caused by the wild-type strain expanded rapidly, and comparable lesion areas were observed on the branches inoculated with complementation strains. However, the Δ*CcSte11* and Δ*CcSte7* caused significantly reduced lesion areas compared to the wild-type strain, which is the same as the Δ*CcPmk1* (Fig. 2).

### CcSte50 is a scaffold of the CcSte11-CcSte7-CcPmk1 cassette

In *S. cerevisiae*, *B. cinerea* and *F. graminearum*, Ste50 is regarded as the scaffold protein of Ste11-Ste7-Kss1 pathway and plays important roles in vegetative growth or virulence (Lee and Elion 1999; Schamber et al. 2010; Gu et al. 2015a). Based on the sequence of Ste50 (AAA13629.1) in *S. cerevisiae*, FgSte50 (jgi|Fusgr1|3949|FGSG_04101T0) in *F. graminearum* and BcSte50 (jgi|Botci1|10,142|BC1T_07505) in *B. cinerea*, we identified CcSte50 (GME6632_g) in *C. chrysosperma*. CcSte50 encodes a 494 aa protein and contains SAM domain and RA domain (Fig. S4).

**Fig. 3:**
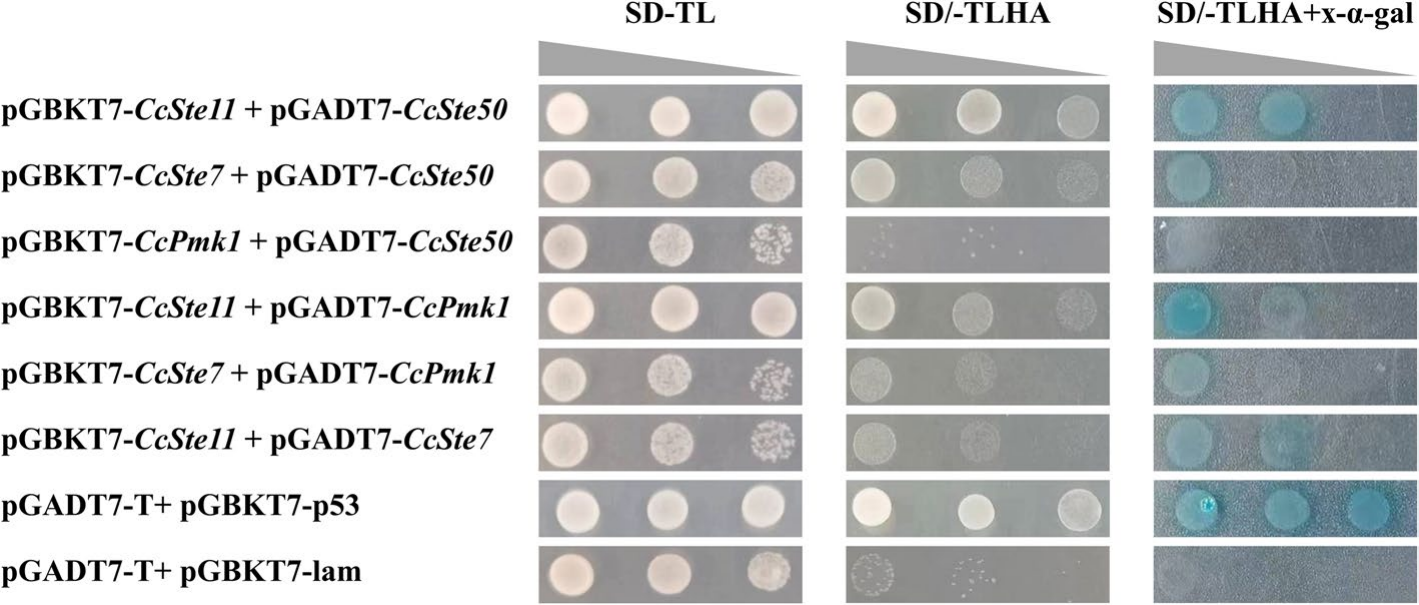
The adaptor CcSte50 interact with CcSte11 and CcSte7. The CcPmk1-MAPKs physically interact with each other

Further, we tested the interactions of CcSte50 with three MAPKs of the Pmk1 pathway using the yeast two-hybrid assays. As shown in Fig. 3, CcSte50 physically interacted with CcSte11 and CcSte7, but not with CcPmk1. Moreover, the different degrees of interaction were detected between any pair of CcSte11, CcSte7 and CcPmk1. Thus, this complex consists of the three kinases CcSte11, CcSte7 and CcPmk1, as well as the adaptor protein CcSte50.

### Homology analysis of *CcSte12*

**Fig. 4:**
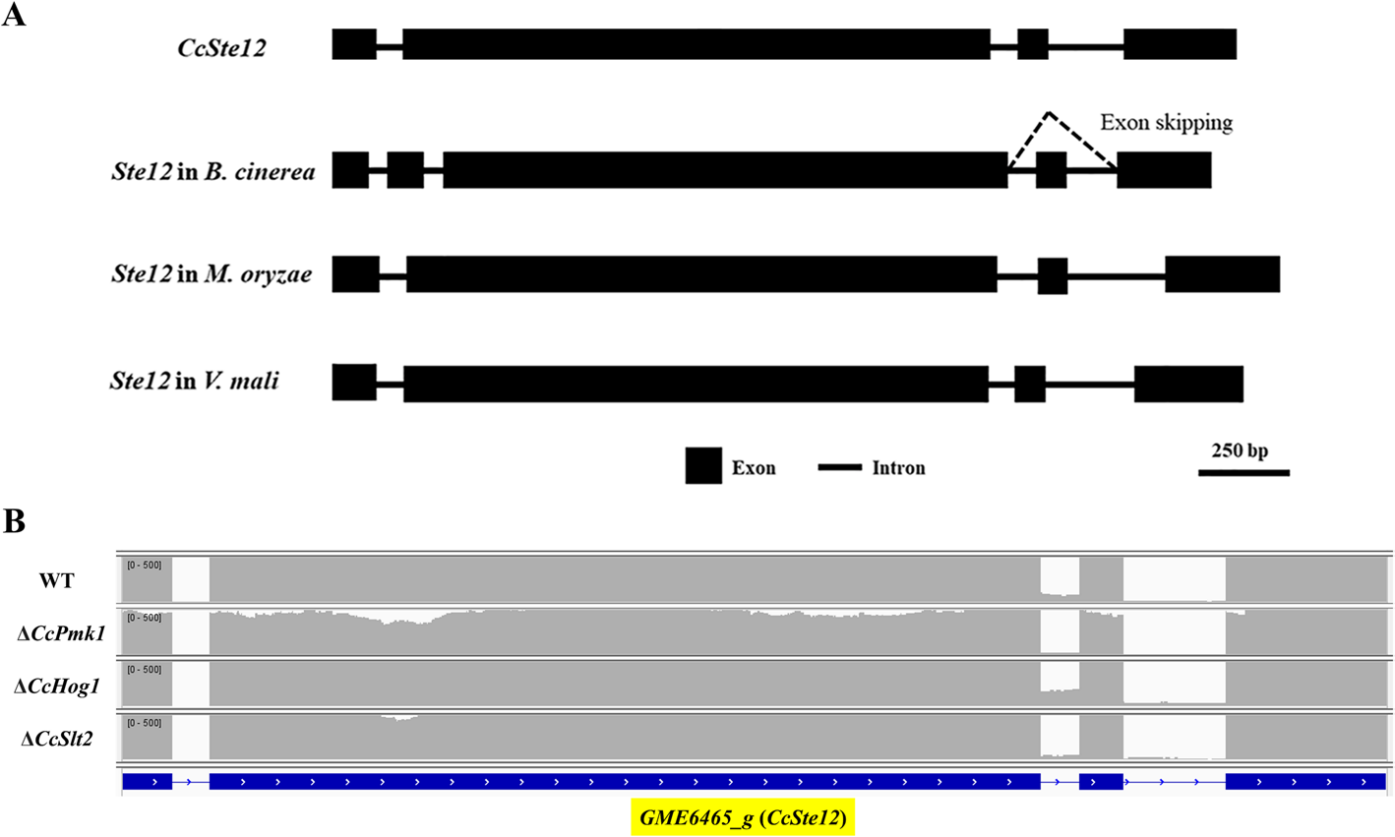
The alternative splicing of *CcSte12*. **A** Gene structure of the *CcSte12* and its orthologs. The boxes represent the exons, the connected lines represent intron. **B** The transcriptional sequencing reads of *CcSte12* in Δ*CcPmk1*, Δ*CcHog1* and Δ*CcSlt2*

The above results revealed the shared functions of *CcSte11*, *CcSte7* and *CcPmk1* in development and pathogenicity and the previous studies exhibited *CcPmk1* acts as a core regulator of fungal pathogenicity by modulating a small number of master downstream targets, such as *CcSte12*. CcSte12 is also highly homologous to other Ste12 orthologs from other fungi and shares 80% identity with Mst12 in *M. oryzae*, 85% identity with CpSte12 in *C.parasitica* and 78% identity with FgSte12 in *F. graminearum* (Fig. S5 and Tab. S2). In *B. cinerea*, two splice variants of *BcSte12* transcripts have been described (Schamber et al. 2010). Intriguingly, the *CcSte12* shared similar gene structure as several other *Ste12* orthologs (Fig. 4A) and alterative splicing transcripts (the intron retention events) were found in *CcSte12* among different samples (*CcPmk1* deletion mutant, *CcHog1* deletion mutant and *CcSlt2* deletion mutant) during the simulated infection process (Fig. 4B).

### Overview of RNA-seq data between wild-type and Δ*CcSte12*

To explore the global regulation network of *CcSte12*, we conducted comparative transcriptional analysis between *CcSte12* deletion mutants and wild-type strain during the simulated infection process.

A total of approximate 260 million reads with an average of about 43,526,636 reads in each sample, ranging from 41,414,452 to 44,679,158 reads, were obtained (Tab. S3). In addition, over 90% of the reads in each sample were mapped to the draft genome of *C. chrysosperma*, indicating the high abundance and excellent quality of the sequencing data (Tab. S3). Principal component analysis (PCA) of read counts of the wild-type and *CcSte12* deletion mutants revealed a clear separation of the two tested conditions and the proximity of biological replicates (Fig. S6A). Moreover, the distributions of gene expression value were comparable among all samples (Fig. S6B and C).

Gene expression analysis showed a total of 10,177/10227 (99.51%) and 10,151/10227 (99.26%) predicted genes were detected in the wild-type and *CcSte12* deletion mutants respectively (Tab. S3). Among these, a total of 10101predicted genes were detected in both the wild-type and Δ*CcSte12*, while 76 and 50 predicted genes were specifically expressed in the wild-type and Δ*CcSte12* (Tab. S3 and Fig. S6D). Differentially expressed genes (DEGs) analysis revealed that 746 predicted genes were significantly down-regulated and 496 were significantly up-regulated in the Δ*CcHog1* compared with the wild type, 1242 DEGs in total (Fig. S6E).

### Functional analysis of differentially expressed genes

**Fig. 5:**
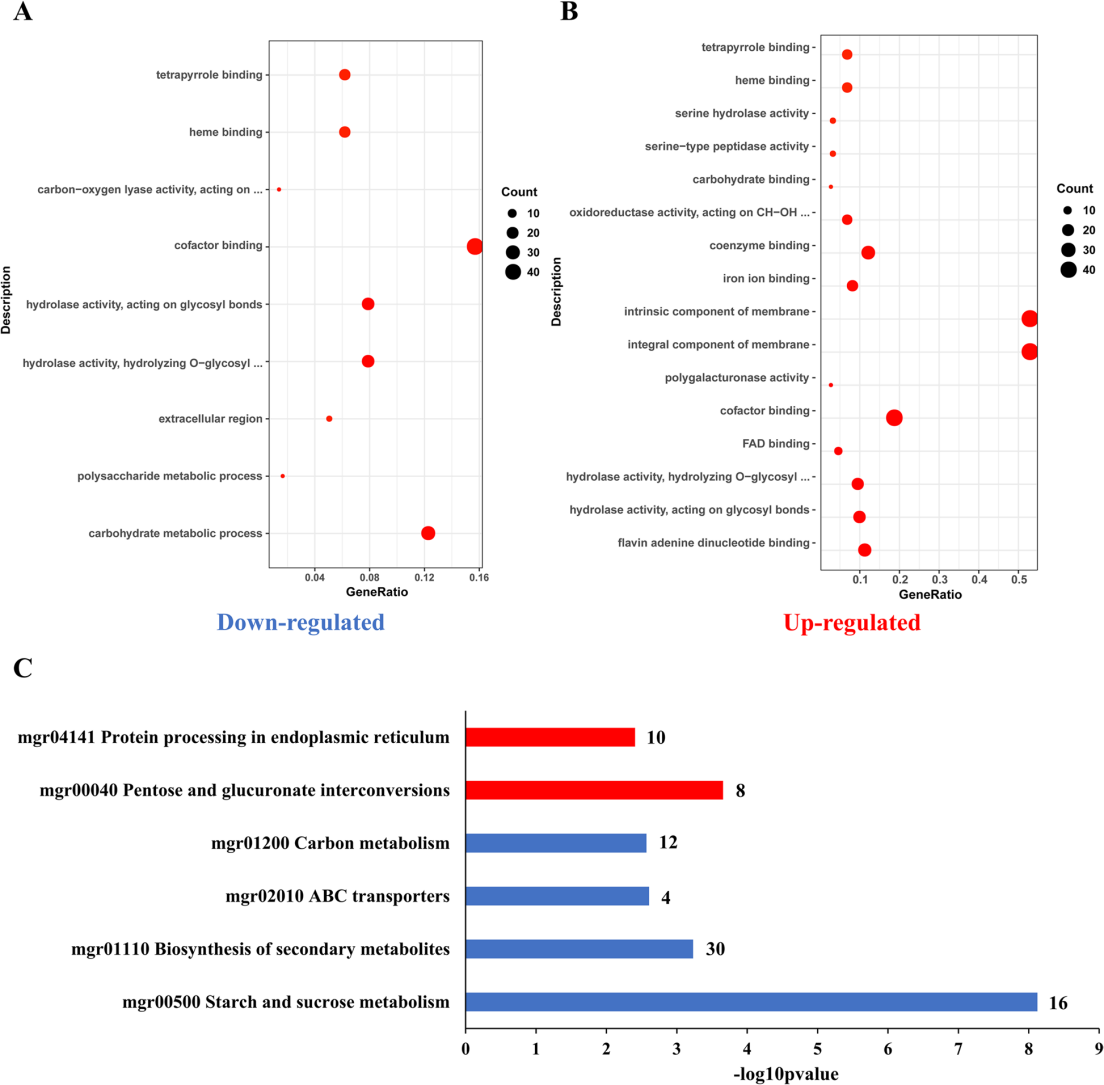
Significant enrichment data between wild-type and Δ*CcSte12* (*p* < 0.01). **A** GO significant enrichment of significantly down-regulated genes in the *CcSte12* deletion mutant compared with wild type. **B** GO significant enrichment of significantly up-regulated genes in *CcSte12* deletion mutant compared with wild type. **C** KEGG pathway significant enrichment of DEGs in Δ*CcSte12* compared with wild-type. The red bars represent the KEGG significant enrichment of up-regulated DEGs, and the blue bars represent the KEGG significant enrichment of down-regulated DEGs. The numbers on the bars represent number of genes enrichment in each KEGG pathway among the DEGs

Gene ontology (GO) enrichment analysis of significantly down-regulated DEGs in Δ*CcSte12* revealed nine significantly enriched terms, including six Molecular Function (MF) terms, two Biological Processes (BP) terms and one Cellular Component (CC) term. The CC term is associated with extracellular region and two BP terms are related to carbohydrate metabolic (Fig. 5A and Tab. S4). There were three terms related to binding functions and three terms are connected with catalytic activity in significant enrichment MF terms. Especially, two terms associated with hydrolase activity (GO:0004553 and GO:0016798) and one term: carbon-oxygen lyase activity (GO:0016838) were in catalytic activity. On the contrary, GO analysis revealed significant enrichment of up-regulated DEGs of Δ*CcSte12* in the two CC and fourteen MF categories (Fig. 5B and Tab. S4). Flavin adenine dinucleotide binding was the dominant enriched term in MF. And, integral component of membrane and intrinsic component of membrane were significantly enriched CC terms.

Kyoto Encyclopedia of Genes and Genomes (KEGG) enrichment analysis revealed four significantly enriched KEGG pathways in the down-regulated DGEs of Δ*CcSte12*, including ABC transporters (mgr02010) and three pathways related to metabolism: starch and sucrose metabolism (mgr00500), biosynthesis of secondary metabolites (mgr01110) and carbon metabolism (mgr01200). In addition, pentose and glucuronate interconversions, and protein processing in endoplasmic reticulum were enriched significantly in up-regulated DEGs of Δ*CcSte12* (Fig. 5C and Tab. S4).

**Fig. 6:**
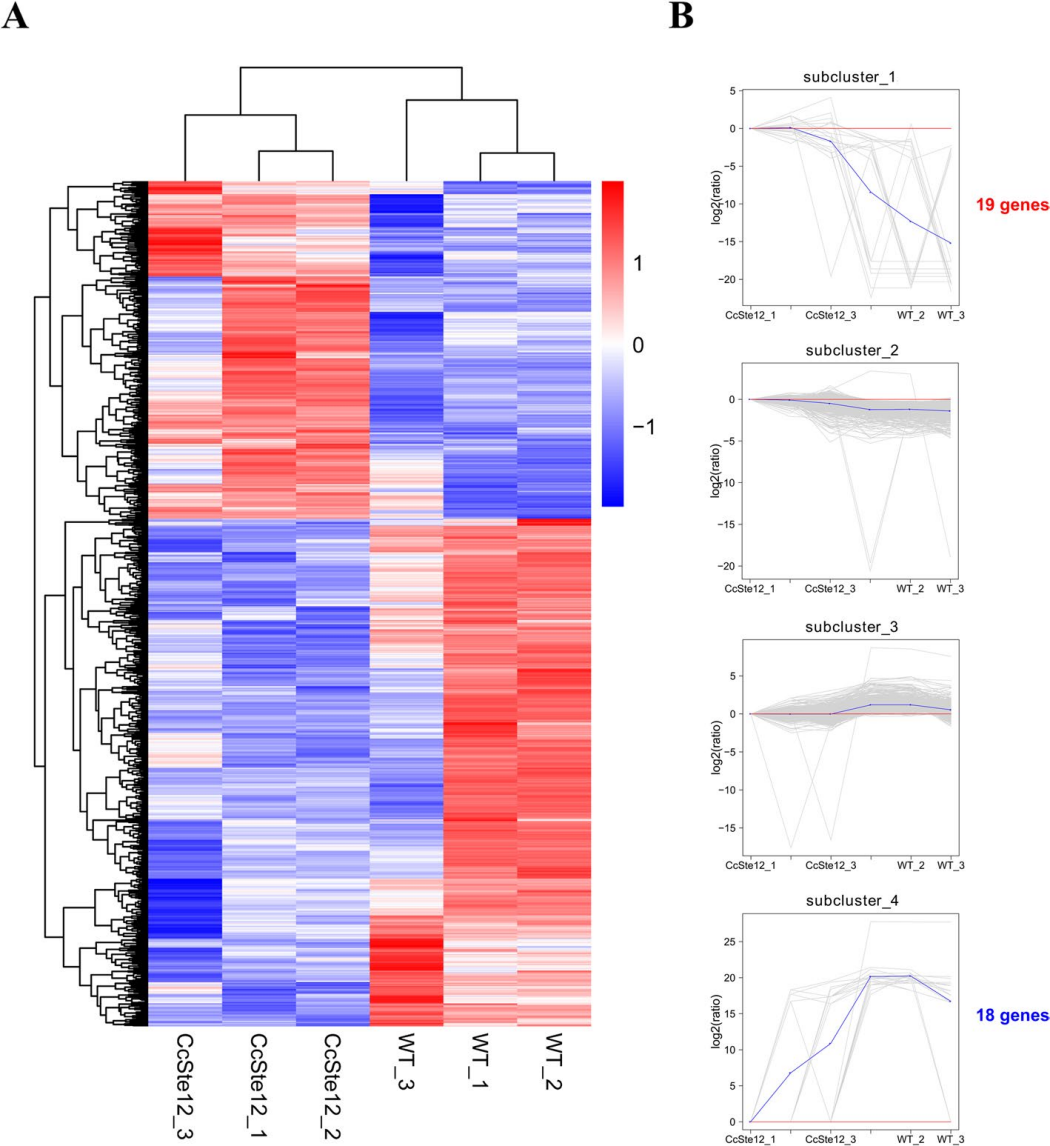
Gene expression pattern of differentially expressed genes. **A** The heatmap shows the expression level in the *CcSte12* deletion mutant and wild type. Genes showing similar patterns of gene expression are clustered. **B** Gene expression analysis according to the expression level. The number of genes in cluster 1 and cluster 4 is showing

All DEGs were visualized in a heat map, created using log2(FPKM+ 1) and then normalized the data by Z-score treatment. Genes showing similar patterns of expression were grouped together by hierarchical clustering (Fig. 6A). Three wild-type and *CcSte12* deletion mutant repeats were cluster together respectively, indicating the high quality of sequencing samples. Fuzzy clustering analysis of differentially expressed genes generated four clusters. The major differentially expressed genes were belonged to cluster 2 and cluster 3 with mild up- or down- regulation, while the cluster 1 contained 19 genes that were dramatically increased their expression levels in *CcSte12* deletion mutant compared with wild-type (Fig. 6B). In addition, the cluster 4 contained 18 genes that were dramatically reduced their expression levels in *CcSte12* deletion mutant (Fig. 6B). Among them, seven genes were annotated to enzymes, including four oxidoreductases and three hydrolases: *GME9493_g* and *GME2503_g* annotated to Glycosyl hydrolases and *GME9508_g* annotated to Beta-1,3-glucanase (Table 1).

**Table 1:** List of differentially expressed genes in Cluster 4

Gene ID	Gene length (bp)	Gene description	Pfam_family
*GME6465_g*	2022	Transcription factor steA; STE like transcription factor	STE
*GME9493_g*	1143	Glycoside-hydrolase family GH114	–
*GME9576_g*	1632	Viridicatumtoxin synthesis protein L; Major Facilitator Superfamily	MFS_1
*GME6217_g*	162	–	–
*GME6677_g*	237	–	–
*GME9508_g*	1383	Beta-1,3-glucanase	–
*GME7584_g*	1572	Cytochrome P450 monooxygenase; Apicidin F synthesis protein 7	p450
*GME9873_g*	714	Glutathione-dependent formaldehyde-activating enzyme	–
*GME3415_g*	1164	–	–
*GME5205_g*	1395	–	–
*GME5370_g*	747	BTB/POZ domain	BTB
*GME2503_g*	1686	Probable rhamnogalacturonase A; Glycosyl hydrolases family 28	–
*GME5863_g*	2376	Cellobiose dehydrogenase; Cellobiose-quinone oxidoreductase; Cytochrome domain of cellobiose dehydrogenase; oxidoreductase	–
*GME5803_g*	1209	–	–
*GME10454_g*	1080	Cytochrome P450 monooxygenase; Tropolone synthesis protein D	p450
*GME9316_g*	1536	–	–
*GME9858_g*	306	–	–
*GME3343_g*	918	–	–

### *CcSte12* regulated the expression of glycosyl hydrolases and effectors

**Fig. 7:**
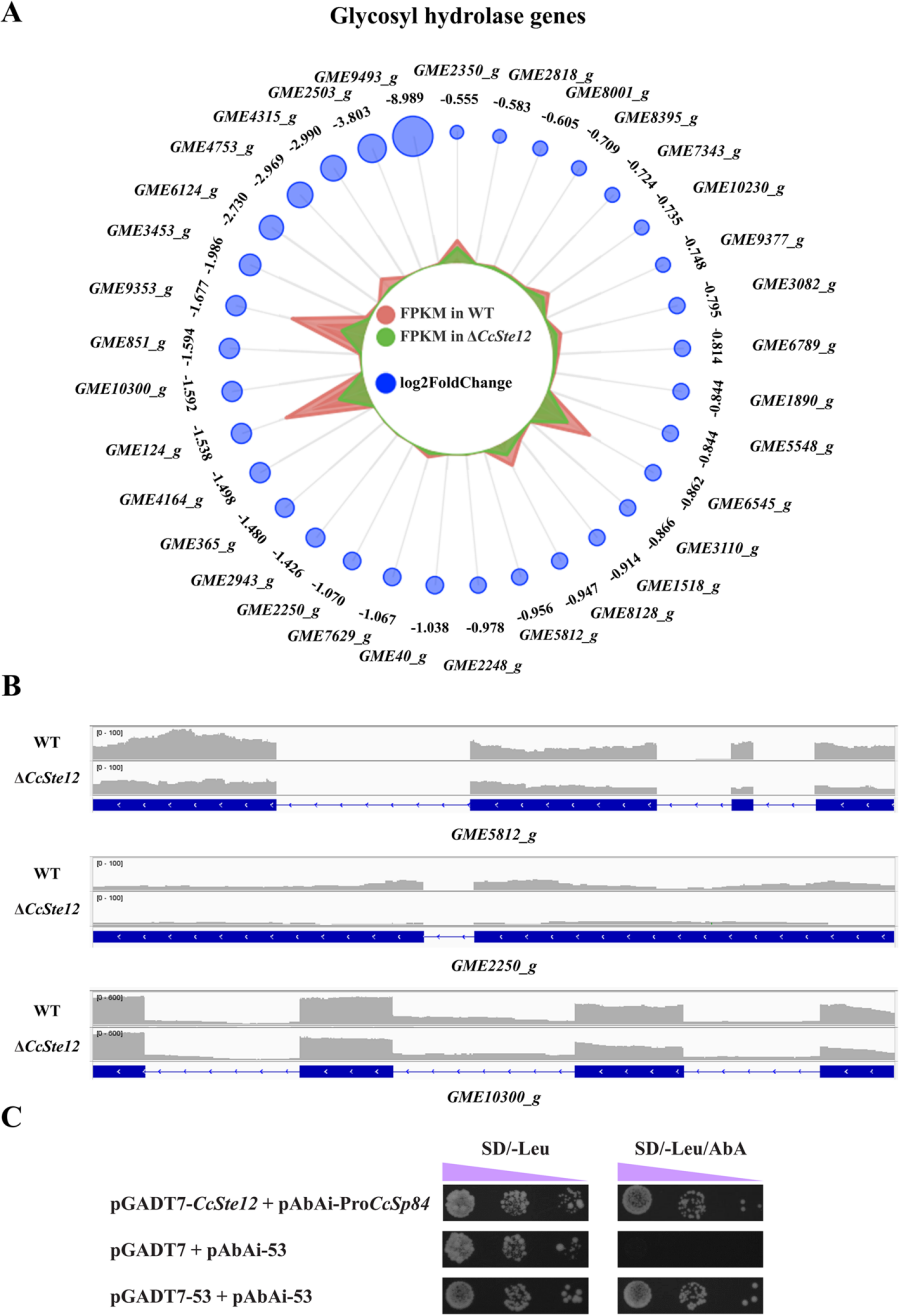
Glycoside hydrolases were regulated by *CcSte12*. **A** The radar chart showed expression profiles of down-regulated glycoside hydrolase genes in Δ*CcSte12* compared to the wild-type. The glycoside hydrolase genes ID are displayed in the outermost circle. The penultimate circle of numbers represents log2(foldchange), which visualized by the blue circle. The red and green areas in the circle indicated the FPKM of glycoside hydrolase genes in wild-type and Δ*CcSte12*, respectively. **B** Three glycoside hydrolase 12 family genes (*GME8128_g*, *GME2250_g* and *GME10300_g*) were significantly down-regulated in Δ*CcSte12*. The gray curves indicate read coverage for the samples from the RNA-Seq. **C** Y1H assay on binding of CcSte12 protein to the promoter region of CcSp84. Purple triangle represents the range of yeast concentrations (10^0^ to 10^−3^). pGADT7-p53 + pAbAi-p53 was used as a positive control and pGADT7 + pAbAi-p53 as a negative control

According to the 18 genes listed in Table 1, we found two glycoside hydrolases and a glucanase. Further investigation revealed that there were 55 hydrolases significantly down-regulated expression in *CcSte12* deletion mutant, including 33 Glycosyl hydrolases (GH) (Fig. 7A). These down-regulated GHs covered 21 GH families, most of which contained only one or two down-regulated members. While, GH3 family, GH5 family and GH12 family contained three down-regulated GHs. In particular, *GME8128_g*, *GME2250_g* and *GME10300_g* were annotated to GH12 family genes (Fig. 7B), and *GME10300_g* was XEG1 homolog. XEG1 from *Phytophthora sojae*, which exhibits xyloglucanase activity and promotes infection by degrading plant cell walls (Ma et al. 2015). Additionally, *GME8128_g*, a GH61 family gene, has been characterized in *C. chrysosperma* and was named *CcSp84*, which was essential for fungal pathogenicity (Xu et al. 2022). Importantly, *CcSp84* contains the element TGAAACA in its promoter, which is a sequence interaction with the Ste12 protein in *S. cerevisiae* (Yuan and Fields 1991). Hence, a yeast one-hybrid (Y1H) assay was conducted to examine the interaction between *CcSte12* and the *CcSp84*-promoter. The results showed that the *CcSte12* binds to the promoter region containing the TGAAACA motif (Fig. 7C).

**Fig. 8:**
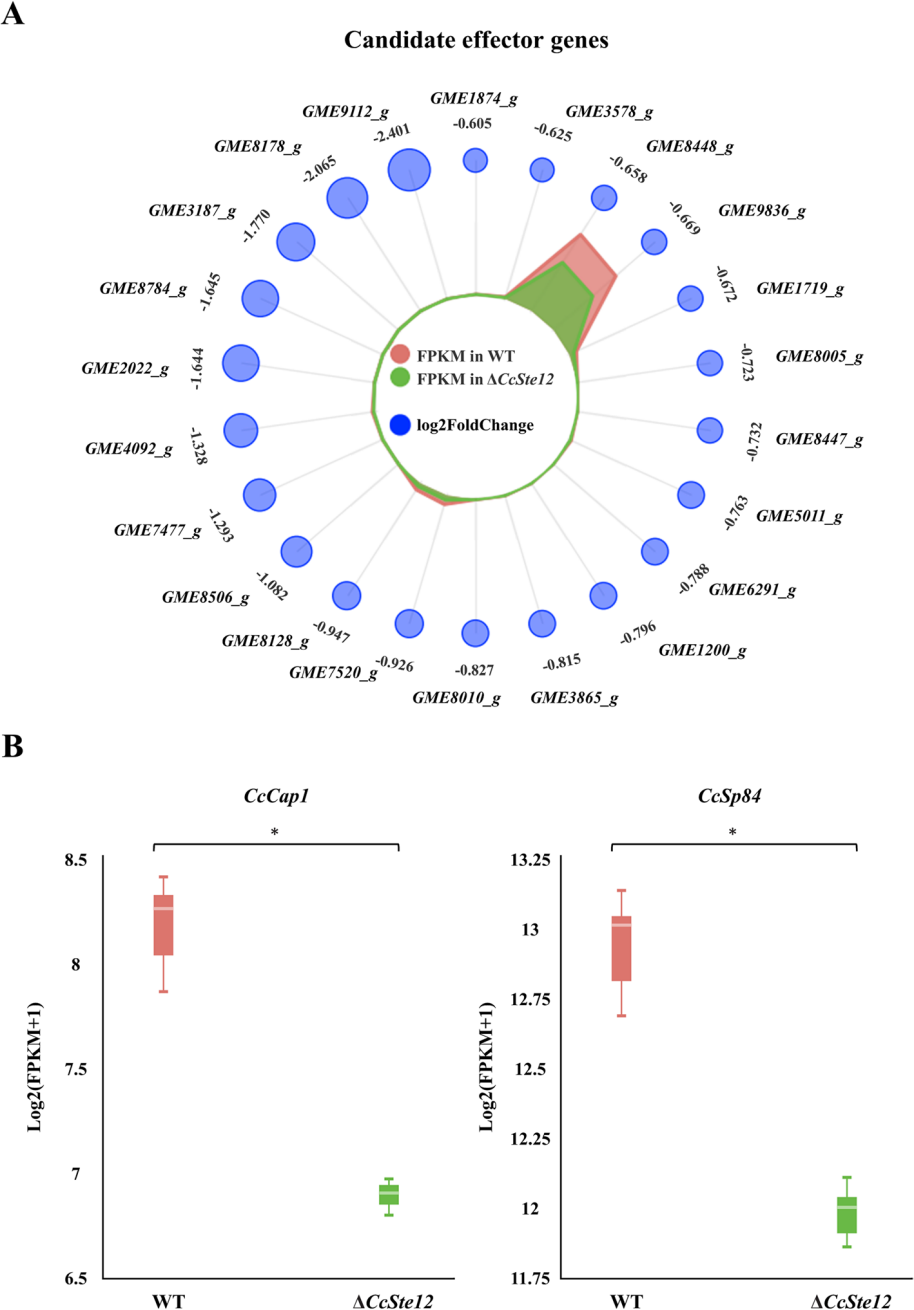
Candidate effector genes showed significantly reduced expression levels in Δ*CcSte12*. **A** The radar chart showed expression profiles of down-regulated candidate effector genes in Δ*CcSte12* compared to the wild-type. The candidate effector genes ID are displayed in the outermost circle. The penultimate circle of numbers represents log2(foldchange), which visualized by the blue circle. The red and green areas in the circle indicated the FPKM of candidate effector genes in wild-type and Δ*CcSte12*, respectively. **B** The expression of two effector (CcCap1 and CcSp84) in wild-type and Δ*CcSte12*

Besides, *CcSp84* is also an effector and could trigger plant immunity. Moreover, previous works in *M. oryzae* have shown that *Mst12* regulates a subset of effectors involved in suppression of host immunity (Oses-Ruiz et al. 2021). Here, we systematically analyzed the candidate effector genes regulated by the *CcSte12*, and found 22 down-regulated putative effectors (Fig. 8A). Among them, there are 4 candidate effector genes (*GME2022_g*, *GME4092_g*, *GME9112_g* and *CcSp84*) contains TGAAACA motif in their promoters. In addition to *CcSp84*, the cysteine-rich secretory protein family gene, *CcCap1* (*GME7477_g*) (Fig. 8B), had been shown to be important for *C. chrysosperma* pathogenicity and could disturb the plant immunity (Han et al. 2021). Furthermore, *GME9836_g* was annotated as cerato-ulmin hydrophobin family and could act as a secreted toxin hydrophobin and might be involved in contact and communication between the fungus and its environment (Temple et al. 1997; Whiteford and Spanu 2001). And *GME8447_g* was annotated as GDSL esterases and lipases. Interestingly, these four candidate effector genes also regulated by *CcPmk1* as well (Yu et al. 2023).

### Comparative transcriptome analysis between *CcSte12* and *CcPmk1* regulated genes

**Fig. 9:**
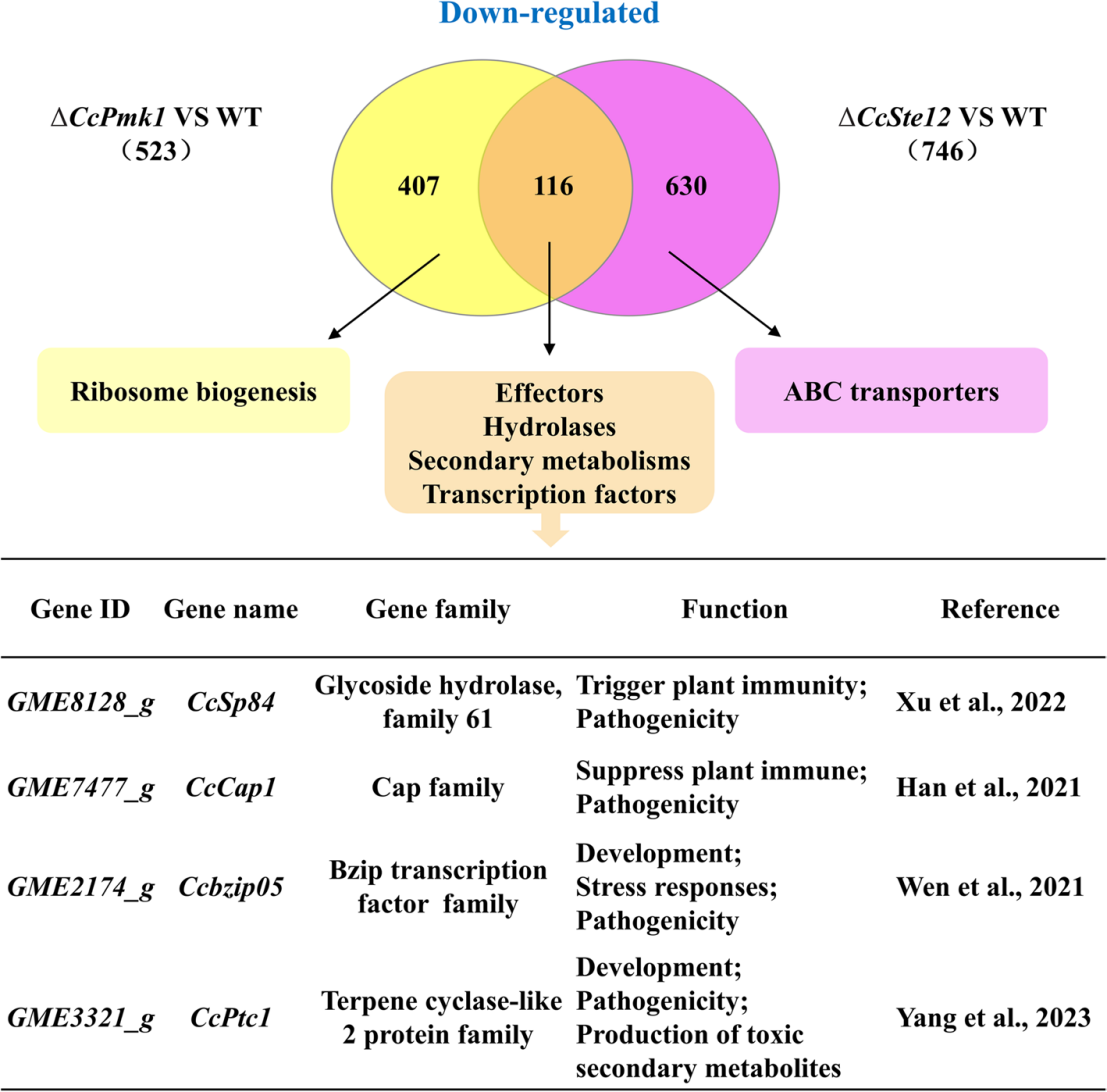
Comparative transcriptomic analysis of *CcSte12*-regulated genes and *CcPmk1*-regulated genes. Venn diagrams showing the number of genes controlled by *CcSte12* and *CcPmk1*. Common significantly down-regulated genes in orange box, including putative effectors, hydrolases, putative secondary metabolite core biosynthesis genes and some transcription factors. Among them, some published virulence-related genes in *C. chrysosperma* were list. The yellow box indicated genes only regulated by *CcPmk1* and the violet box indicated genes only regulated by *CcSte12*

In above results, we found some genes regulated by *CcSte12* also reduced their expression in *CcPmk1* deletion mutants. Therefore, differentially expressed genes in the *CcSte12* deletion mutants and *CcPmk1* deletion mutants were analyzed. Comparisons of gene expression exhibited 116 genes were overlapping down-regulated DEGs in *CcSte12* deletion mutants and *CcPmk1* deletion mutants. Among them, we found 7 effectors, 10 hydrolases, 6 putative secondary metabolite core biosynthesis genes and some transcription factors (Fig. 9). Moreover, some of them has been reported to be associated with virulence, including *GME3321_g* (*CcPtc1*), *GME2174_g* (*CcBzip05*), *CcCap1* and *CcSp84* (Fig. 9). *Ccbzip05* is a Bzip transcription factor and required for fungal development, stress responses and pathogenicity (Wen et al. 2021). *GME3321_g* (*CcPtc1*) is terpene cyclase-like 2 protein family members and is important for virulence, mycotoxin production and development (Yang et al. 2023). In addition, 407 and 630 genes down-regulated only in the *CcPmk1* deletion mutant and *CcSte12* deletion mutant, respectively. Genes associated with ribosome processing were not expressed in the *CcSte12* deletion mutants and *CcSte12* regulated ABC transporters not with *CcPmk1* (Fig. 9).

To confirm the validity of the transcriptomic data, we performed qRT-PCR analysis of some DEGs described above, including three glycosyl hydrolases (*GME5812_g*, *GME2250_g* and *GME10300_g*), two effectors (*CcCap1* and *CcSp84*) and two genes associated with virulence (*CcPtc1* and *CcBzip05*). The expression levels of these genes were significantly down-regulated in the *CcSte12* deletion mutants compared with the wild type (Fig. S7). Consequently, the expression patterns of these genes in the qRT-PCR analysis were basically consistent those detected in the RNA-sequencing analysis, indicating that the transcriptome data were accurate and reliable.

## Discussion

The MAPK cascade pathway is located in the center of the cell signal transmission network, and is involved in regulating cell growth and differentiation, metabolism, adaptation to adverse environment, infection of pathogens, and other physiological processes (Li et al. 2022). Kss1 and Fus3 are two partially redundant MAPK pathways in *S. cerevisiae* that have overlapping functions in pheromone response and filamentation or invasive growth into agar (Morillon et al. 2000). By comparison, most ascomycetous fungi have only one Fus3/Kss1 ortholog pathway, which plays an important role in regulating the pathogenicity of pathogenic fungi. Hence, this pathway is also known as Pmk1-MAPK pathway (Zhang et al. 2021b). In plant pathogenic fungi, this conserved MAPK cascade is best characterized in *M. oryzae*. The *Pmk1* of *M. oryzae* contributes to fungal virulence by regulating the formation of appressoria, activating fungal effector genes expression and controlling the constriction of invasive hyphae at plasmodesmata sites (Sakulkoo et al. 2018). Similar to the *Pmk1* mutant, the *Mst7* and *Mst11* deletion mutants were nonpathogenic and failed to form appressoria (Zhao et al. 2005). Thus, appressorium formation and plant infection may the dominant mechanism regulated by the Pmk1-MAPK pathway of *M. oryzae*. This mechanism is also found in CgMK1-MAPK pathway of *C. gloeosporioides*. Deletion of *CgSte50*, *CgSte11*, *CgSte7* and *CgMk1* induced similar defects in appressorium formation, polar growth, mycelium melanin, the abiotic stress response and pathogenicity (Wang et al. 2021). By contrast, the Pmk1-MAPK pathway is usually associated with secondary metabolism in *Fusarium* species. For example, Gpmk1 regulates the early induction of extracellular endoglucanase, xylanolytic, and proteolytic activities and is responsible for the overall induction of secreted lipolytic activities in *F. graminearum* (Ren et al. 2019; Yang et al. 2022). And deletion of *FgSte11* and *FgSte7*, results in similar defects with gpmk1 mutant in development and pathogenicity (Ramamoorthy et al. 2007). In our study, deletion of *CcSte11*, *CcSte7* and *CcPmk1* induced similar defects in fungal development and pathogenicity. These results indicate that the components of CcPmk1-MAPK share conserved functions in virulence.

Our research displayed that CcSte11, CcSte7 and CcPmk1 interact with each other, and similar results were observed in *Aspergillus* species. AnSte11-AnSte7-AnFus3 form a physically interacting module that is required for sexual development in *Aspergillus nidulans* (Bayram et al. 2012). The SteC (Ste11) -MkkB (Ste7) -MpkB (Fus3), physically nteract and regulates development, stress responses and secondary metabolism in *Aspergillus fumigatus* (Dean Frawley et al. 2020). While, in *M. oryzae*, no direct interaction was detected between Pmk1 and Mst7 or Mst11 (Zhao et al. 2005). The direct interaction between Mst7 and Pmk1 was detected only during appressorium formation, and Mst11 weekly interacted with Mst7 (Zhao et al. 2005; Zhao and Xu 2007). N-terminal region of Mst11 directly interacted with its kinase domain, and its self-inhibitory binding is important for regulation of kinase activity (Qi et al. 2015). Anyway, Pmk1 is activated by Mst7 and Mst11, and like other fungi, *M. oryzae* lacks a distinct homolog of yeast scaffold protein Ste5. Instead, Mst50 functions as an adaptor protein that interacts with Mst11 and Mst7 as well as Ras proteins (Zhao and Xu 2007; Zhou et al. 2014) In addition, the thioredoxin Trx2 plays a role in proper folding and interaction with Mst7 and regulates the activation of Pmk1 MAPK through its interaction with Mst7 (Zhang et al. 2016). In the current study, CcSte50 also interacts with CcSte11 and CcSte7, not CcPmk1. Therefore, the mechanisms that modulate the activity of the Pmk1 MAPK cascade among fungi is convergent and distinct.

Once the Pmk1-MAPKs complexes assembled, kinase phosphorylation enables transduction of a signal downstream, via translocation of Pmk1 into the nucleus where it interacts with various transcription factors such as Ste12 (Hamel et al. 2012). Our previous research revealed that CcPmk1 could phosphorylate and interact with the downstream homeobox transcription factor CcSte12 by phosphoproteomic analyses and yeast two-hybrid assays (Yu et al. 2022). Phosphoproteomic analyses between the CcPmk1 deletion mutant and wild type showed the phosphorylation level of CcSte12 was significantly reduced in the *CcPmk1* deletion mutant compared to the wild type. In addition, the abundance of peptides that included the phosphorylated residues CcSte12^Ser405^, CcSte12^Ser487^, and CcSte12^Ser545^ was significantly reduced in the *CcPmk1* deletion mutant compared to the wild type. Moreover, the phosphorylated residue CcSte12^Ser405^ harbored a MAPK S/T-P phosphorylation motif. While, deletion of *CcPmk1* significantly reduced but did not abolish the phosphorylation of CcSte12 (Yu et al. 2022), indicating that CcSte12 is also phosphorylated by kinase proteins other than CcPmk1 and CcPmk1 has some additional downstream transcription factors. In addition, the Δ*CcSte12* displayed similar defect phenotypes as Δ*CcPmk1* but not the same, such as significantly reduced fungal growth, conidiation, and virulence (Yu et al. 2022). Consistent with this finding, we found some genes regulated by *CcSte12* also reduced their expression in *CcPmk1* deletion mutants, while a number of genes are regulated only by *CcPmk1* or *CcSte12*. Therefore, *CcSte12* may not only function downstream from *CcPmk1* and *CcPmk1* has some additional downstream transcription factors.

In pathogenic fungi, Ste12 and Ste12-like proteins play a critical role in fungal development and pathogenicity (Wong Sak Hoi and Dumas 2010). For example, *B. cinerea* lacking Ste12 showed normal germination, but delayed infection as a result of low penetration efficiency (Schamber et al. 2010). And two differently spliced ste12 transcripts were detected, and both were able to complement the ste12 mutant, except for a defect in sclerotium formation (Schamber et al. 2010). Additionally, the exon skipping events was described in Ste12 ortholog in *C. lindemuthianum*, which lead to the two transcribed versions of Ste12 (Wong Sak Hoi et al. 2007). Impartantly, both of them truncated the C2H2 zinc finger and the abundance of two Ste12 splicing variants were dynamically changing during different stages. Intriguingly, the exon skipping region of Ste12 was highly conserved in different fungi, but the same alternative splicing of Ste12 was not found so far in other phytopathogenic fungi. CcSte12 contains Home-Ste domain at N terminus and two tandem C_2_H_2_ zinc fingers at C terminus (Yu et al. 2022). In this study, we also calculated the putative splicing variants of *CcSte12* and alterative splicing transcripts (the intron retention events in two tandem C_2_H_2_ zinc fingers) were found in *CcSte12* among different samples (*CcPmk1* deletion mutant, *CcHog1* deletion mutant and *CcSlt2* deletion mutant) during the simulated infection process, indicating that the alternative splicing of Ste12 might universal. Interestingly, our previous data showed *CcSte12* is significantly down-regulated in Δ*CcPmk1* only, and the expression level of *CcSte12* was no significant change in Δ*CcHog1* or Δ*CcSlt2* (Yu et al. 2023). While, these kinases regulated the alternative splicing of *CcSte12*, positively or negatively. How these kinases affect the alternative splicing events of *CcSte12* remains unclear. And the presence of the alternative RNA of CcSte12 is too low, whose functions is mystery. These are worth exploring in the future.

To define the regulatory mechanism of *CcSte12* in virulence of *C. chrysosperma*, we used RNA-seq to investigate downstream genes regulated by *CcSte12*. The RNA-seq analysis revealed two hydrolase activity GO terms (GO:0004553 and GO:0016798) and starch and sucrose metabolism (mgr00500) KEGG pathway were significantly enriched in the down-regulated genes of *CcSte12* deletion mutants. In addition, a subset of hydrolase genes was significantly down-regulated in the *CcSte12* deletion mutant, especially glycoside hydrolase, such as *GME10300_g* (glycoside hydrolase 12 protein EG1 homology) and *CcSp84* (glycoside hydrolase 61 protein). Glycoside hydrolase 12 (GH12) family proteins have been systematically characterized in *F. graminearum* and Glycoside hydrolase GH12 family genes *Fg05851* and *Fg11037* act redundantly to regulate *F. graminearum* virulence (Wang et al. 2023). EG1 homology acted as a pathogen-associated molecular pattern (PAMP) targeting the apoplast of plants to induce cell death. For example, *FoEG1*, a secreted glycoside hydrolase family 12 protein from *F. oxysporum*, triggers cell death and modulates plant immunity (Zhang et al. 2021a). In addition, *FoEG1* contributes to the pathogenicity of *F. oxysporum* as well. Moreover, *CcSp84* was critical for fungal pathogenicity and could also induce cell death and immune responses in N. *benthamiana* (Xu et al. 2022). And the plant nucleus localization of *CcSp84* was essential to trigger the plant defense responses (Xu et al. 2022). Importantly, *CcSp84* contain the element TGAAACA in its promoter and *CcSte12* interacts with the *CcSp84*-promoter. In *S. cerevisiae*, a pheromone response element (PRE) containing a sequence of TGAAACA is able to interact with the Ste12 protein (Yuan and Fields 1991). The STE domain has also been found to be required for DNA binding in *Cryptococcus neoformans* (Chang et al. 2004) and the sequence of TGAAACA recognized by Ste12 is important for binding in *C. lindemuthianum* (Wong Sak Hoi et al. 2007). In *F. graminearum*, *FgSte12* regulated four cellulase genes contained this element in their promoter regions, which may be important for pathogenicity. Because, deletion of *FgSte*12 does not affect the DON biosynthesis, unlike the gpmk1 mutant. Although FgSte12 interacts with the FgSte11–Ste7–Gpmk1 complex and FgSte12 is regulated by Gpmk1 kinase (Gu et al. 2015a; Gu et al. 2015b).

A recent report exhibited that Mst12 regulated a subset of effectors involved in suppression of host immunity in *M. oryzae* (Oses-Ruiz et al. 2021). Besides, *CfSte12* of *Colletotrichum fructicola* also regulated effectors in early pathogenesis (Liu et al. 2021). Effectors facilitate pathogen infection by interfering with host defenses and modulating plant immunity. In our study, 22 candidate effector genes down regulated in *CcSte12* deletion mutants. Interestingly, 7 of them also regulated by *CcPmk1*. In addition to *CcSp84*, the cysteine-rich secretory protein family gene, *CcCap1* has been described *C. chrysosperma* (Han et al. 2021). *CcCap1* localizes to both the plant nucleus and cytoplasm, and is essential and sufficient for its suppression activity of the PTI response (Han et al. 2021). Furthermore, we identified genes require Pmk1 or Mst12 and found *CcPtc1* and *CcBzip05* down-regulated in *CcSte12* deletion mutants and *CcPmk1* deletion mutants. *CcPtc1* acts as a virulence-related secondary metabolism factor and *CcBzip05* is important for fungal pathogenicity (Yang et al. 2023; Wen et al. 2021). Pathogenic fungi have evolved sophisticated ways to infect their hosts, mainly by adapting to the host environment and producing pathogenesis-related products such as toxins, extracellular enzymes, effectors, and/or toxic secondary metabolites (Jonkers et al. 2012). Effectors can disturb the plant immunity and promote pathogenic processes (Presti et al. 2015), and secondary metabolism is crucial determinants of plant disease (Richard 2007). *C. chrysosperma* is a necrotrophic forest pathogenic fungus that infects woody plants through micro-wounds and no typical infection structure has been found so far. Thus, producing pathogenesis-related products, such as toxins, extracellular enzymes, effectors, and/or toxic secondary metabolites, may be more important to infect its hosts. Therefore, *CcSte12* may participate in regulating the expression of a subset of downstream components, especially hydrolases and effectors to promote virulence.

## Conclusion

**Fig. 10:**
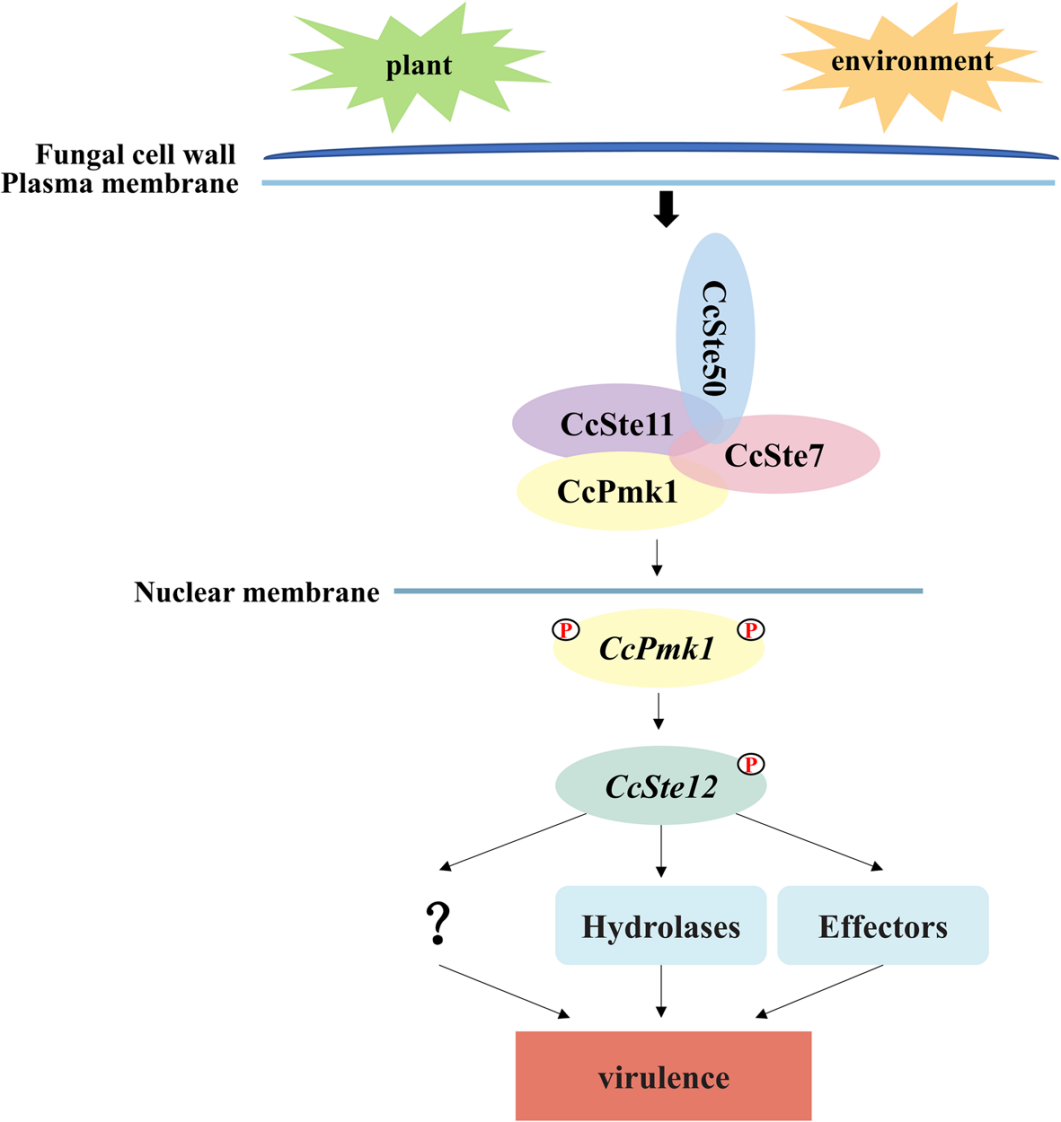
Proposed model for the role of CcPmk1-MAPKs pathway in virulence of *C. chrysosperma*. Cell surface sensors recognize a variety of host and environment signals and activate the CcPmk1-MAPK pathway. CcSte11, CcSte7 and CcPmk1 can form protein complexes, which receive signals transmitted by upstream adaptor CcSte50. *CcPmk1* translocate into the nucleus, where it interacts with transcription factors *CcSte12* to positively regulate hydrolase, effectors and other genes to promote virulence. “P” represents phosphate groups

In conclusion, the CcPmk1-MAPK signaling pathway of *C. chrysosperma* plays a key role in the pathogenicity. CcSte11, CcSte7 and CcPmk1 interact with each other, and form protein complexes, which receive signals transmitted by upstream CcSte50 and transmit them to downstream CcSte12, which regulates hydrolase enzymes and other genes to promote virulence (Fig. 10).

## Materials and methods

### Fungal strains and culture conditions

*Cytospora chrysosperma* strain CFCC 89981 was used as the wild-type strain for constructing various gene deletion mutants. The *CcPmk1* deletion mutants were created in our previous studies (Yu et al. 2019). The wild-type strain, resultant gene deletion and complemented strains were cultured at 25°C on potato dextrose agar (PDA) medium routinely. Liquid yeast extract peptone dextrose (YEPD) medium and potato dextrose broth (PDB) medium were used for the shaking culture of the conidia and mycelia respectively, in order to protoplast preparation and transformation. Transformants were incubated in TB3 medium supplemented with antibiotics for selecting mutants.

### Sequence analyses

The sequences of CcSte50, CcSte11 and CcSte7 were obtained from the genome database of *C. chrysosperma* (tel. *Valsa sordida*), NCBI GenBank accession number JAEQMF000000000, which is sequenced by our laboratory. BLAST searches of homologs from other microorganisms were performed using NCBI and JGI websites (https://www.ncbi.nlm.nih.gov and https://mycocosm.jgi.doe.gov/mycocosm/home) with E_value < 10^−6^. Protein domain predictions were analyzed using InterPro website (http://www.ebi.ac.uk/interpro/). The amino acid sequence alignments were performed using ClustalW and visualized with Jalview. The phylogenetic tree was constructed with MEGA 10.0 using the neighbour-joining method with the bootstrap test replicated 1000 times.

### Construction of gene deletion and complementation mutants

The split-marker methods were used to generate the targeted deletion cassette (Catlett et al. 2003; Goswami 2012). The upstream and downstream flanking sequences of target gene and the hygromycin B resistance cassette (HPH), including overlap sequence with the flanking sequences, was amplified. Then, the upstream and downstream fragments and two-thirds of the hygromycin cassette are fused together, respectively. For the complementation of the target gene, a fragment containing the entire length of the target gene along with its native promoter and terminator regions was amplified. All fragments were confirmed by sequencing analysis.

The protoplast preparation and PEG-mediated transformation were conducted as described (Yu et al. 2019). Targeted gene was replaced with the hygromycin cassette and complementation of the mutants by co-transformation with the geneticin resistance cassette. Transformants were analyzed by PCR amplification with specific primers and Southern blot to verify homologous insertion of the construct. All primers used in this study were described in Tab. S5.

### Development analysis and pathogenicity tests

The 5 mm mycelium plugs from the edge of a growing colony were used to inoculate new PDA plates, which were cultivated at 25°C in the dark. Colony shape and colour were observed and the colony diameters were measured daily. For testing conidial production, the number of pycnidia per plate was measured after culture incubation under 60-day/night cycles. All assays were repeated three times.

For the pathogenicity assay, detached annual poplar twigs with similar growth tendency and thickness from the same test field were prepared. Then, the twigs were cut into 20 cm-long segments, of which the ends were sealed with wax, after washing with distilled water, 75% ethanol and 1% sodium hypochlorite. Each twig segment was wounded using a flat iron (five mm-diameter). After the scorch treatment, the wounded samples were inoculated with mycelium agar (5 mm diameter) obtained from the edge of the wild-type, gene deletion mutants, and complemented strains on PDA. The inoculated twigs were placed in trays, and the trays were covered with parafilm to retain humidity. Then, the inoculated twigs were cultured at 25°C under day/night cycles with proper humidity. Lesions of inoculated twigs were photographed and measured at different time intervals. All assays were repeated three times, with a minimum of twenty twigs for every treatment.

### Yeast two-hybrid assays

The Matchmaker Gold Yeast Two-Hybrid System (Clontech) was used to assess the protein interaction. The coding sequence regions of each tested gene was amplified from total cDNA of wild-type with primer pairs indicated in Tab. S5. The cDNA of each gene was inserted into vector pGADT7, and vector pGBDKT7, respectively. The test pairs of vectors were co-transformed into yeast strain AH109 following the LiAc/PEG transformation protocol. The interaction between pGBKT7–53 and pGADT7-T was used as the positive control, and the interaction between pGBKT7-Lam and pGADT7-T was used as the negative control. Yeast strains containing both prey and bait were selected on double dropout agar medium (SD-Leu-Trp). The selected yeast strains were then screened on quadruple dropout agar medium (SD-Leu-Trp-His-Ade). The yeast cells were shown with different concentrations (1:1, 1:100, and 1:10000) on the same plate. Transformants were grown at 30°C for 3 days.

### RNA-seq analysis

For the RNA-Seq analysis, the wild-type and the *CcSte12* deletion mutants were grown in PDB supplemented with sterilized poplar twigs to mimic the states of infection, and then incubated at 25°C with shaking at 150 rpm for 2 days. The samples of *CcSte12* deletion mutants were collected in the same condition as CcPmk1 deletion mutants for RNA-seq analysis. Total RNA was extracted from samples using an RNAprep Pure Plant Kit (Tiangen) following the manufacturer’s instructions.

After checking the quantity and quality of RNA, transcriptome samples were prepared using Illumina kits. All cDNA libraries were sequenced on the Illumina HiSeq platform and 150 bp paired-end reads were generated. Raw reads were firstly processed with in-house Perl scripts. In this step, clean reads were obtained by removing reads containing adapter, ploy-N and low quality reads by using the fastp software (fastp -g -q 5 -u 50 -n 15 -l 150) (Chen et al. 2018). Then, paired-end clean reads were aligned to the reference genome using Hisat2 v. 2.0.5 (Kim et al. 2015; Kim et al. 2019).

The number of reads (counts) aligned to each gene was calculated by featureCounts v1.5.0-p3 and quantification of gene expression was based on FPKM (transcript kilobase fragments per million fragments mapped) values. Differentially expressed genes (DEGs) between compared groups were detected using the DESeq2. R package with the following criteria: log2 (fold change) ≥ 0.5; *P* < 0.05. The transcriptome sequencing of wild-type strain was used as the control. The screened DEGs were then evaluated in GO and KEGG enrichment analyses by the clusterProfiler R package. GO and KEGG terms with adjusted *p* < 0.05 were considered significantly enriched by DEGs.

### Quantitative real-time polymerase chain reaction (qRT-PCR) analysis

RT-qPCR analysis was conducted using RNA described above. First-strand cDNA was synthesized from 1 μg RNA with ABScript II cDNA Fist-Strand Synthesis Kit (ABclonal, China), according to the manufacturer’s instructions. The qRT-PCR assay was conducted with 2X Universal SYBR Green Fast qPCR Mix (ABclonal, China) using an ABI 7500 real-time PCR system (Applied Biosystems, USA). The *CcActin* gene served as the endogenous control for all qRT-PCR analyses. All samples were independently subjected to three replicate experiments. The relative expression of genes was calculated by using the 2^−∆∆Ct^ method.

### Yeast one-hybrid assays

The Y1H assay was performed using the Yeast One-Hybrid System-Matchmaker Gold Kit (Clontech) following the manufacturer’s instructions. The full-length of the *CcSte12* gene was ligated to pGADT7 ector (prey plasmid) by homologous recombination, while the target gene promoter fragment was cloned into the pAbAi vector (Bait vector). All primers used in this study were described in Tab. S5. The promoter is defined as 1.5 kb upstream from the transcription initiation site of genes. We cloned a segment containing TGAAACA motif in the promoter of *CcSp84* for Y1H assay to examine the interaction between *CcSte12* and the *CcSp84*-promoter. After linearizing the bait vector plasmids using BstBI, the pAbAi-promoter was transformed into Y1HGold yeast strain and selected on an SD/−Ura plate. The pGADT7-CcSte12 constructs were transformed into strain Y1HGold harboring pAbAi-bait and screened on an SD/−Ura/AbA (Aureobasidin A) plate (200 ng/mL AbA). The pGADT7-p53 + pAbAi-p53 was used as a positive control and pGADT7 + pAbAi-p53 as a negative control. To confirm the results, positive clones (cotransformed) were spotted in serial dilutions of yeast (1:1, 1:10, 1:100, and 1:1000) and cultured on the SD/−Leu/AbA medium at 30°C for 4 days.

### Statistical analysis

Data were biologically repeated three times and represented as means ± standard deviation. The statistical differences between groups were calculated by one-way ANOVA with a Duncan’s range test using SPSS 20.0. *P* < 0.05 was considered as significant differences.

### Supplementary Information

**Additional file 1:** **Figure S1.** Protein structure of CcSte11 and CcSte7, and multiple sequence alignment of Ste11 and Ste7 homologs from different plant pathogenic fungi. The red box represents the conserved DIK motif.

**Additional file 2:** **Figure S2.** Phylogenetic analysis of Pmk1-MAPKs from different plant pathogenic fungi. These fungal Pmk1-MAPK members can be resolved into three separate clades.

**Additional file 3:** **Figure S3.** Deletion and complementation of *CcSte7* and *CcSte11* in *C. chrysosperma*. (A) The Δ*CcSte7* and the complemented strains screening by PCR amplification with specific *CcSte7* and *CcActin* primer pairs. (B) Southern blot analysis of genomic DNA from wild-type and *CcSte7* deletion mutants. Genomic DNA from these isolates was digested with SmaI. The enzyme-digested products were probed with the sequence of hph. (C) The Δ*CcSte11* and the complemented strains screening by PCR amplification with specific *CcSte11* and *CcActin* primer pairs. (D) Southern blot analysis of genomic DNA from wild-type and *CcSte11* deletion mutants. Genomic DNA from these isolates was digested with KpnI. The enzyme-digested products were probed with the sequence of hph.

**Additional file 4:** **Figure S4.** Protein structure of adaptor protein CcSte50, and multiple sequence alignment and phylogenetic analysis of CcSte50 homologs from different plant pathogenic fungi.

**Additional file 5:** **Figure S5.** Multiple sequence alignment and phylogenetic analysis of CcSte12 homologs from different plant pathogenic fungi.

**Additional file 6:** **Figure S6.** Gene expression data among wild type and ΔCcSte12. (A) Principal component analysis of the wild-type and Δ*CcSte12*. (B) The global view of the distribution of gene expression density in the wild-type and Δ*CcSte12*. (C) The global view of the distribution of gene expression level in the wild-type and Δ*CcSte12*. (D) The global view of the number of expressed genes in Δ*CcSte12* and wild-type. (E) The global view of the number of differential expression genes of Δ*CcSte12* and wild-type.

**Additional file 7:** **Figure S7.** The RT-qPCR confirmation of RNA-Seq results. The RT-qPCR analysis was conducted and the expression levels of three glycosyl hydrolases (*GME5812_g*, *GME2250_g* and *GME10300_g*), two effectors (*CcCap1* and *CcSp84*) and two genes associated with virulence (*CcPtc1* and *CcBzip05*) were significantly down-regulated in the *CcSte12* deletion mutants compared with the wild type.

**Additional file 8:** **Table S1.** The detailed information on the CcPmk1-MAPKs homologs and the percentage of identity between CcPmk1-MAPKs and CcPmk1-MAPKs homologues in other fungi.

**Additional file 9:** **Table S2.** RNA-Seq statistics (Sheet 1) and gene expression statistics (Sheet 2).

**Additional file 10:** **Table S3.** The percentage of identity between CcSte12 of *C. chrysosperma* and Ste12 homologs in other fungi.

**Additional file 11:** **Table S4.** GO significant enrichment analysis data and KEGG significant enrichment analysis data. (Sheet 1) GO significant enrichment analysis in DEGs between Δ*CcSte12* and wild type. (Sheet 2) KEGG significant enrichment analysis in DEGs between Δ*CcSte12* and wild type.

**Additional file 12:** **Table S5.** Primers used in this study.

## Data Availability

Not applicable.

## Abbreviations

MAPK: Mitogen-activated protein kinase
MAPKK: Mitogen-activated protein kinase kinase
MAPKKK: Mitogen-activated protein kinase kinase kinase
S or Ser: Serine
T or Thr: Threonine
Y or Tyr: Tyrosine
P or Pro: Proline
D or Asp: Aspartic acid
K or Lys: lysine
SAM: Sterile alpha motif
RA: Ras association
bp: Base pair
aa: Amino acid
PCR: Polymerase chain reaction
PCA: Principal component analysis
DEGs: Differentially expressed genes
GO: Gene ontology
MF: Molecular function
BP: Biological processes
CC: Cellular component
KEGG: Kyoto encyclopedia of genes and genomes
ABC transporter: ATP-binding cassette transporter
FPKM: Reads per kilobase of exon model per million mapped reads
GH: Glycosyl hydrolases
Y1H: Yeast one-hybrid
GDSL: Gly-Asp-Ser-Leu; glycine-aspartic acid- serine-leucine
Bzip: Basic leucine zipper
Y2H: Yeast two-hybrid
PAMP: Pathogen-associated molecular pattern
PRE: Pheromone response element
DON: Deoxynivalenol
PTI: Pattern-triggered immunity
PDA: Potato dextrose agar
PDB: Potato dextrose broth
YEPD: Yeast extract peptone dextrose
WT: Wild-type

## References

[CR1] Asunción García-Sánchez M, Martín-Rodrigues N, Ramos B, de Vega-Bartol JJ, Perlin MH, Díaz-Mínguez JM (2010) *Fost12*, the *Fusarium oxysporum* homolog of the transcription factor Ste12, is upregulated during plant infection and required for virulence. Fungal Genet Biol 47:216–225. 10.1016/j.fgb.2009.11.006 10.1016/j.fgb.2009.11.006 19941968

[CR2] Bayram Ö, Bayram ÖS, Ahmed YL, Maruyama J, Valerius O, Rizzoli SO, Ficner R, Irniger S, Braus GH (2012) The *aspergillus nidulans* MAPK module AnSte11-Ste50-Ste7-Fus3 controls development and secondary metabolism. PLoS Genet 8:e1002816. 10.1371/journal.pgen.1002816 10.1371/journal.pgen.1002816 PMC3400554 22829779

[CR3] Biggs AR, Davis DD, Merrill W (1983) Histopathology of cankers on *Populus* caused by *Cytospora chrysosperma*. Can J Bot 61:563–574. 10.1139/b83-064

[CR4] Catlett NL, Bee-Na L, Yoder OC, Turgeon BG (2003) Split-marker recombination for efficient targeted deletion of fungal genes. Fungal Genet Newsl 50:9–11. 10.4148/1941-4765.1150

[CR5] Chang YC, Wright LC, Tscharke RL, Sorrell TC, Wilson CF, Kwon-Chung KJ (2004). Regulatory roles for the homeodomain and C2H2 zinc finger regions of Cryptococcus neoformans Ste12alphap. Mol Microbiol.

[CR6] Chen S, Zhou Y, Chen Y, Gu J (2018). fastp: an ultra-fast all-in-one FASTQ preprocessor. Bioinformatics.

[CR7] Chou S, Lane S, Liu H (2006). Regulation of mating and filamentation genes by two distinct Ste12 complexes in *Saccharomyces cerevisiae*. Mol Cell Biol.

[CR8] Fan XL, Bezerra JDP, Tian CM, Crous PW (2020). Cytospora (Diaporthales) in China. Persoonia.

[CR9] Frawley D, Stroe MC, Oakley BR, Heinekamp T, Straßburger M, Fleming AB, Brakhage AA, Bayram Ö (2020). The pheromone module SteC-MkkB-MpkB-SteD-HamE regulates development, stress responses and secondary metabolism in *aspergillus fumigatus*. Front Microbiol.

[CR10] Goswami RS (2012). Targeted gene replacement in fungi using a split-marker approach. Methods Mol Biol.

[CR11] Gu Q, Chen Y, Liu Y, Zhang C, Ma Z (2015). The transmembrane protein FgSho1 regulates fungal development and pathogenicity via the MAPK module Ste50-Ste11-Ste7 in *Fusarium graminearum*. New Phytol.

[CR12] Gu Q, Zhang C, Liu X, Ma Z (2015). A transcription factor FgSte12 is required for pathogenicity in *Fusarium graminearum*. Mol Plant Pathol.

[CR13] Hamel LP, Nicole MC, Duplessis S, Ellis BE (2012). Mitogen-activated protein kinase signaling in plant-interacting fungi: distinct messages from conserved messengers. Plant Cell.

[CR14] Han Z, Xiong D, Xu Z, Liu T, Tian C (2021) The *Cytospora chrysosperma* virulence effector CcCAP1 mainly localizes to the plant nucleus to suppress plant immune responses. mSphere 6:e00883–e00820. 10.1128/msphere.00883-20 10.1128/mSphere.00883-20 PMC8544888 33627507

[CR15] Jiang C, Zhang X, Liu H, Xu JR (2018) Mitogen-activated protein kinase signaling in plant pathogenic fungi. PLoS Pathog 14:e1006875. 10.1371/journal.ppat.1006875 10.1371/journal.ppat.1006875 PMC5854419 29543901

[CR16] Jonkers W, Dong Y, Broz K, Kistler HC (2012). The Wor1-like protein Fgp1 regulates pathogenicity, toxin synthesis and reproduction in the phytopathogenic fungus *Fusarium graminearum*. PLoS Pathog.

[CR17] Kepley JB, Reeves FB, Jacobi WR, Adams GC (2015). Species associated with *cytospora* canker on *Populus tremuloides*. Mycotaxon.

[CR18] Kim D, Langmead B, Salzberg SL (2015). HISAT: a fast spliced aligner with low memory requirements. Nat Methods.

[CR19] Kim D, Paggi JM, Park C, Bennett C, Salzberg SL (2019). Graph-based genome alignment and genotyping with HISAT2 and HISAT-genotype. Nat Biotechnol.

[CR20] Lee BN, Elion EA (1999). The MAPKKK Ste11 regulates vegetative growth through a kinase cascade of shared signaling components. Proc Natl Acad Sci U S A.

[CR21] Li L, Zhu XM, Zhang YR, Cai YY, Wang JY, Liu MY, Wang JY, Bao JD, Lin FC (2022). Research on the molecular interaction mechanism between plants and pathogenic Fungi. Int J Mol Sci.

[CR22] Liu W, Liang X, Gleason LM, Cao M, Zhang R (2021). Transcription factor *CfSte12* of *Colletotrichum fructicola* is a key regulator of early apple glomerella leaf spot pathogenesis. Appl Environ Microbiol.

[CR23] Presti LL, Lanver D, Schweizer G, Tanaka S, Liang L, Tollot M (2015). Fungal effectors and plant susceptibility. Annu Rev Plant Biol.

[CR24] Ma Z, Song T, Zhu L, Ye W, Wang Y, Shao Y (2015). A *Phytophthora sojae* glycoside hydrolase 12 protein is a major virulence factor during soybean infection and is recognized as a PAMP. Plant Cell.

[CR25] Madhani HD, Fink GR (1997). Combinatorial control required for the specificity of yeast MAPK signaling. Science.

[CR26] Madhani, H. D. and Cora A. Styles (1997) MAP Kinases with distinct inhibitory functions impart signaling specificity during yeast differentiation. 10.1016/s0092-8674(00)80454-7 10.1016/s0092-8674(00)80454-7 9393860

[CR27] Morillon A, Springer M, Lesage P (2000). Activation of the Kss1 invasive-filamentous growth pathway induces Ty1 transcription and retrotransposition in Saccharomyces cerevisiae. Mol Cell Biol.

[CR28] Oses-Ruiz M, Cruz-Mireles N, Martin-Urdiroz M, Soanes DM, Eseola AB, Tang B (2021). Appressorium-mediated plant infection by *Magnaporthe oryzae* is regulated by a Pmk1-dependent hierarchical transcriptional network. Nat Microbiol.

[CR29] Park G, Bruno KS, Staiger CJ, Talbot NJ, Xu JR (2004). Independent genetic mechanisms mediate turgor generation and penetration peg formation during plant infection in the rice blast fungus. Mol Microbiol.

[CR30] Park G, Xue C, Zhao X, Kim Y, Orbach M, Xu JR (2006). Multiple upstream signals converge on the adaptor protein Mst50 in *Magnaporthe grisea*. Plant Cell.

[CR31] Park G, Xue C, Zheng L, Lam S, Xu JR (2002). *MST12* regulates infectious growth but not appressorium formation in the rice blast fungus *Magnaporthe grisea*. Mol Plant-Microbe Interact.

[CR32] Qi L, Kim Y, Jiang C, Li Y, Peng Y, Xu JR (2015). Activation of Mst11 and feedback inhibition of germ tube growth in *Magnaporthe oryzae*. Mol Plant-Microbe Interact.

[CR33] Ramamoorthy V, Zhao X, Snyder AK, Xu JR, Shah DM (2007). Two mitogen-activated protein kinase signalling cascades mediate basal resistance to antifungal plant defensins in *Fusarium graminearum*. Cell Microbiol.

[CR34] Ren J, Li C, Gao C, Xu JR, Jiang C, Wang G (2019). Deletion of *FgHOG1* is suppressive to the *mgv1* mutant by stimulating *Gpmk1* activation and avoiding intracellular turgor elevation in *Fusarium graminearum*. Front Microbiol.

[CR35] Richard JL (2007). Some major mycotoxins and their mycotoxicoses--an overview. Int J Food Microbiol.

[CR36] Rispail N, Di Pietro A (2009). *Fusarium oxysporum* Ste12 controls invasive growth and virulence downstream of the Fmk1 MAPK cascade. Mol Plant-Microbe Interact.

[CR37] Sakulkoo W, Osés-Ruiz M, Oliveira Garcia E, Soanes DM, Littlejohn GR, Hacker C, Correia A, Valent B, Talbot NJ (2018) A single fungal MAP kinase controls plant cell-to-cell invasion by the rice blast fungus. Science 359:1399–1403. 10.1126/science.aaq0892 10.1126/science.aaq0892 29567712

[CR38] Schamber A, Leroch M, Diwo J, Mendgen K, Hahn M (2010). The role of mitogen-activated protein (MAP) kinase signalling components and the Ste12 transcription factor in germination and pathogenicity of *Botrytis cinerea*. Mol Plant Pathol.

[CR39] Temple B, Horgen PA, Bernier L, Hintz WE (1997). Cerato-ulmin, a hydrophobin secreted by the causal agents of dutch elm disease, is a parasitic fitness factor. Fungal Genet Biol.

[CR40] Tsuji G, Fujii S, Tsuge S, Shiraishi T, Kubo Y (2003). The Colletotrichum lagenariu Ste12-like gene CST1 is essential for appressorium penetration. Mol Plant-Microbe Interact.

[CR41] Turra D, Segorbe D, Di Pietro A (2014). Protein kinases in plant-pathogenic fungi: conserved regulators of infection. Annu Rev Phytopathol.

[CR42] Wang X, Lu D, Tian C (2021). Mitogen-activated protein kinase cascade CgSte50-Ste11-Ste7-Mk1 regulates infection-related morphogenesis in the poplar anthracnose fungus *Colletotrichum gloeosporioides*. Microbiol Res.

[CR43] Wang Z, Yang B, Zheng W, Wang L, Cai X, Yang J (2023). Recognition of glycoside hydrolase 12 proteins by the immune receptor RXEG1 confers *Fusarium* head blight resistance in wheat. Plant Biotechnol J.

[CR44] Wen D, Yu L, Xiong D, Tian C (2021). Genome-wide identification of bZIP transcription factor genes and functional analyses of two members in *Cytospora chrysosperma*. J Fungi.

[CR45] Whiteford JR, Spanu PD (2001). The hydrophobin HCf-1 of *Cladosporium fulvum* is required for efficient water-mediated dispersal of conidia. Fungal Genet Biol.

[CR46] Wong Sak Hoi J, Dumas B (2010). Ste12 and Ste12-like proteins, fungal transcription factors regulating development and pathogenicity. Eukaryot Cell.

[CR47] Wong Sak Hoi J, Herbert C, Bacha N, O'Connell R, Lafitte C, Borderies G (2007). Regulation and role of a STE12-like transcription factor from the plant pathogen *Colletotrichum lindemuthianum*. Mol Microbiol.

[CR48] Xiong D, Yu L, Shan H, Tian C (2021). *CcPmk1* is a regulator of pathogenicity in *Cytospora chrysosperma* and can be used as a potential target for disease control. Mol Plant Pathol.

[CR49] Xu JR, Hamer JE (1996). MAP kinase and cAMP signaling regulate infection structure formation and pathogemc growth in the rice blast fungus *Magnaporthe grisea*. Gene Dev.

[CR50] Xu Z, Xiong D, Han Z, Tian C (2022). A putative effector CcSp84 of *Cytospora chrysosperma* localizes to the plant nucleus to trigger plant immunity. Int J Mol Sci.

[CR51] Yang Y, Huang P, Ma Y, Jiang R, Jiang C, Wang G (2022). Insights into intracellular signaling network in *Fusarium* species. Int J Biol Macromol.

[CR52] Yang Y, Yu L., Xiaolin Q, Xiong D and Tian C (2023) A putative terpene cyclase gene (CcPtc1) is required for fungal development and virulence in *Cytospora chrysosperma.*Front Microbiol 14:1084828. 10.3389/fmicb.2023.1084828 10.3389/fmicb.2023.1084828 PMC9986285 36891381

[CR53] Yu L, Wen D, Yang Y, Qiu X, Xiong D, Tian C (2023). Comparative transcriptomic analysis of MAPK-mediated regulation of pathogenicity, stress responses and development in *Cytospora chrysosperma*. Phytopathology.

[CR54] Yu L, Xiong D, Han Z, Liang Y, Tian C (2019). The mitogen-activated protein kinase gene *CcPmk1* is required for fungal growth, cell wall integrity and pathogenicity in *Cytospora chrysosperma*. Fungal Genet Biol.

[CR55] Yu L, Yang Y, Xiong D, Tian C (2022) Phosphoproteomic and Metabolomic profiling uncovers the roles of CcPmk1 in the pathogenicity of Cytospora chrysosperma. Microbiol Spectrum 10:e0017622. 10.1128/spectrum.00176-22 10.1128/spectrum.00176-22 PMC9430611 35735975

[CR56] Yuan YL, Fields S (1991). Properties of the DNA-binding domain of the Saccharomyces cerevisiae STE12 protein. Mol Cell Biol.

[CR57] Zhang L, Yan J, Fu Z, Shi W, Ninkuu V, Li G (2021). FoEG1, a secreted glycoside hydrolase family 12 protein from *Fusarium oxysporum*, triggers cell death and modulates plant immunity. Mol Plant Pathol.

[CR58] Zhang S, Jiang C, Zhang Q, Qi L, Li C, Xu JR (2016). Thioredoxins are involved in the activation of the PMK1 MAP kinase pathway during appressorium penetration and invasive growth in *Magnaporthe oryzae*. Environ Microbiol.

[CR59] Zhang X, Wang Z, Jiang C, Xu J-R (2021). Regulation of biotic interactions and responses to abiotic stresses by MAP kinase pathways in plant pathogenic fungi. Stress Biol.

[CR60] Zhao X, Kim Y, Park G, Xu JR (2005). A mitogen-activated protein kinase cascade regulating infection-related morphogenesis in *Magnaporthe grisea*. Plant Cell.

[CR61] Zhao X, Mehrabi R, Xu JR (2007). Mitogen-activated protein kinase pathways and fungal pathogenesis. Eukaryot Cell.

[CR62] Zhao X, Xu JR (2007). A highly conserved MAPK-docking site in Mst7 is essential for Pmk1 activation in *Magnaporthe grisea*. Mol Microbiol.

[CR63] Zhou X, Zhao X, Xue C, Dai Y, Xu JR (2014). Bypassing both surface attachment and surface recognition requirements for appressorium formation by overactive ras signaling in *Magnaporthe oryzae*. Mol Plant-Microbe Interact.

