# Supplementary figures and images for: The mitogen-activated protein kinase module CcSte11-CcSte7-CcPmk1 regulates pathogenicity via the transcription factor CcSte12 in Cytospora chrysosperma
Source: Stress Biol. 2024 Jan 16;4(1):4. doi: 10.1007/s44154-023-00142-w (PMC10789715; doi:10.1007/s44154-023-00142-w)

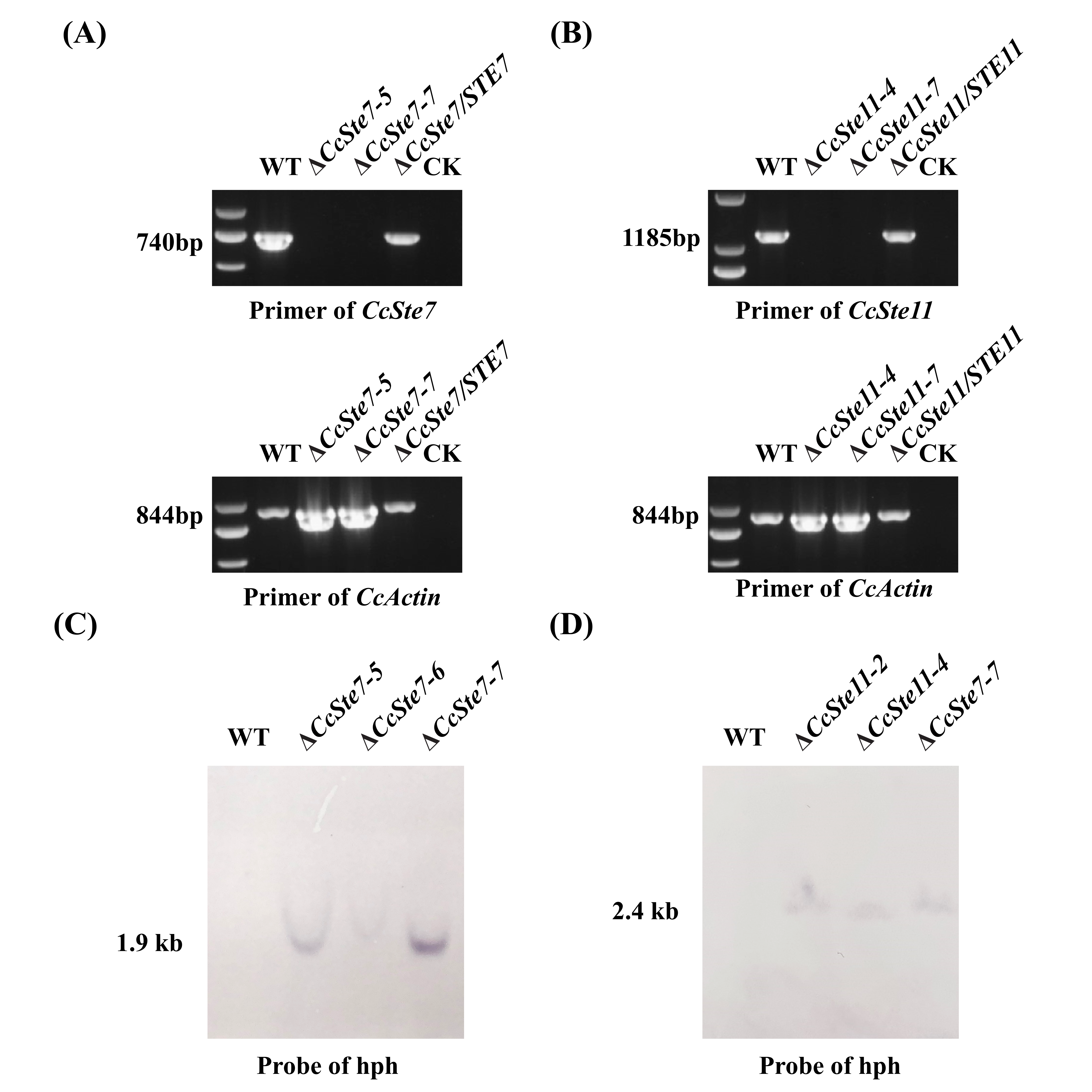

Supplement: Supplementary file 3 — Additional file 3: Figure S3. Deletion and complementation of CcSte7 and CcSte11 in C. chrysosperma. (A) The ΔCcSte7 and the complemented strains screening by PCR amplification with specific CcSte7 and CcActin primer pairs. (B) Southern blot analysis of genomic DNA from wild-type and CcSte7 deletion mutants. Genomic DNA from these isolates was digested with SmaI. The enzyme-digested products were probed with the sequence of hph. (C) The ΔCcSte11 and the complemented strains screening by PCR amplification with specific CcSte11 and CcActin primer pairs. (D) Southern blot analysis of genomic DNA from wild-type and CcSte11 deletion mutants. Genomic DNA from these isolates was digested with KpnI. The enzyme-digested products were probed with the sequence of hph. [file 44154_2023_142_MOESM3_ESM.tif]

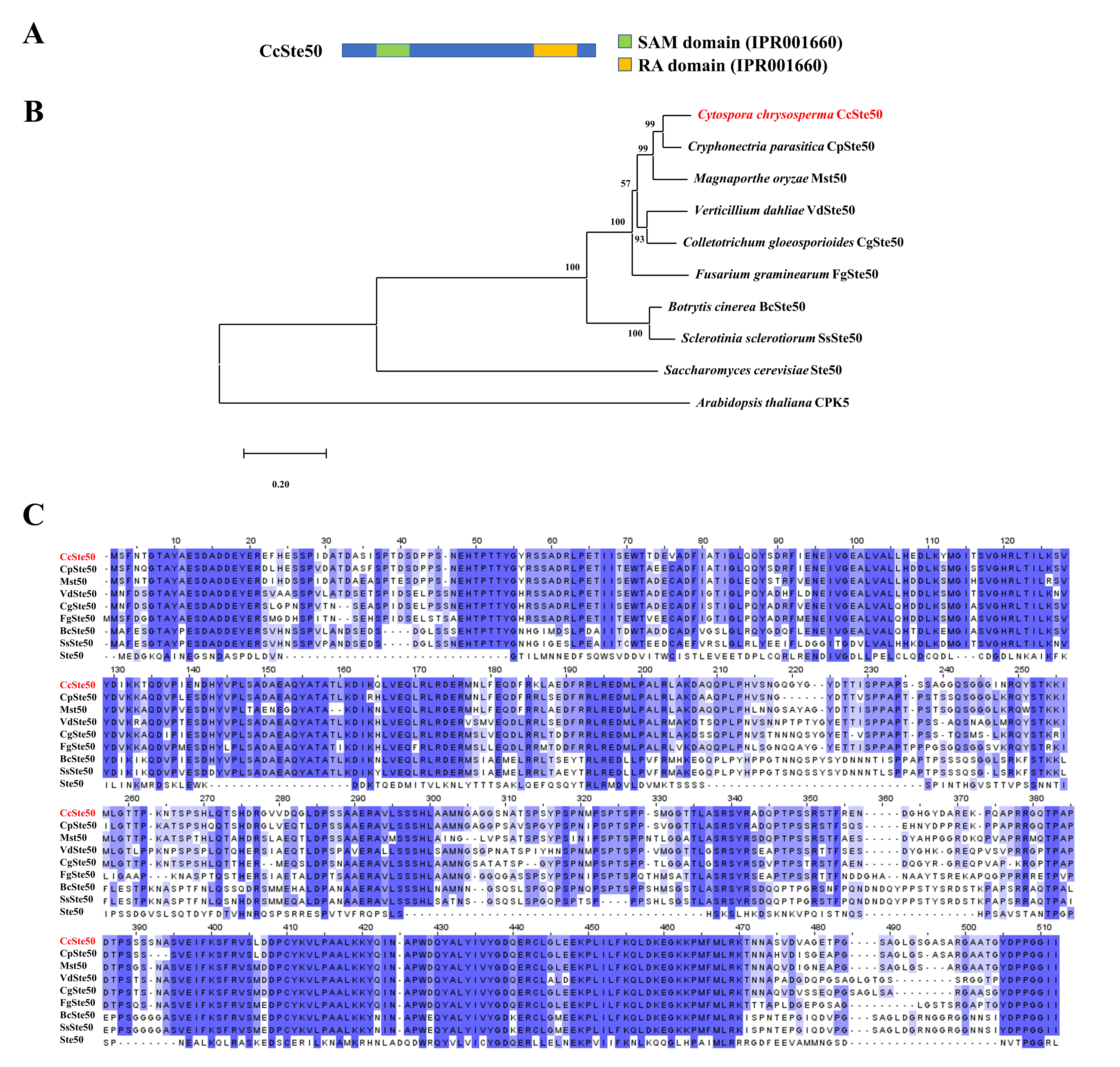

Supplement: Supplementary file 4 — Additional file 4: Figure S4. Protein structure of adaptor protein CcSte50, and multiple sequence alignment and phylogenetic analysis of CcSte50 homologs from different plant pathogenic fungi. [file 44154_2023_142_MOESM4_ESM.tif]

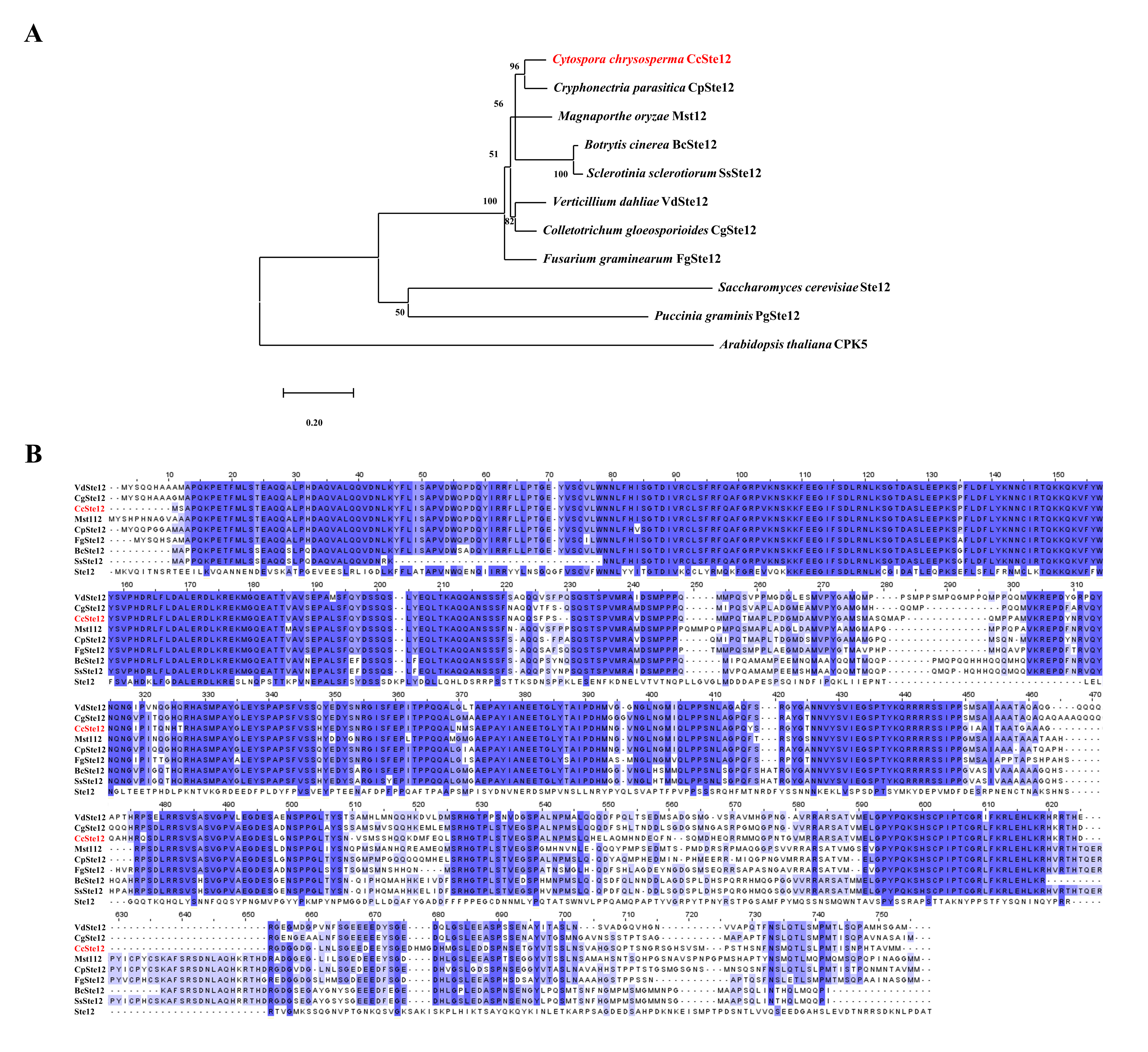

Supplement: Supplementary file 5 — Additional file 5: Figure S5. Multiple sequence alignment and phylogenetic analysis of CcSte12 homologs from different plant pathogenic fungi. [file 44154_2023_142_MOESM5_ESM.tif]

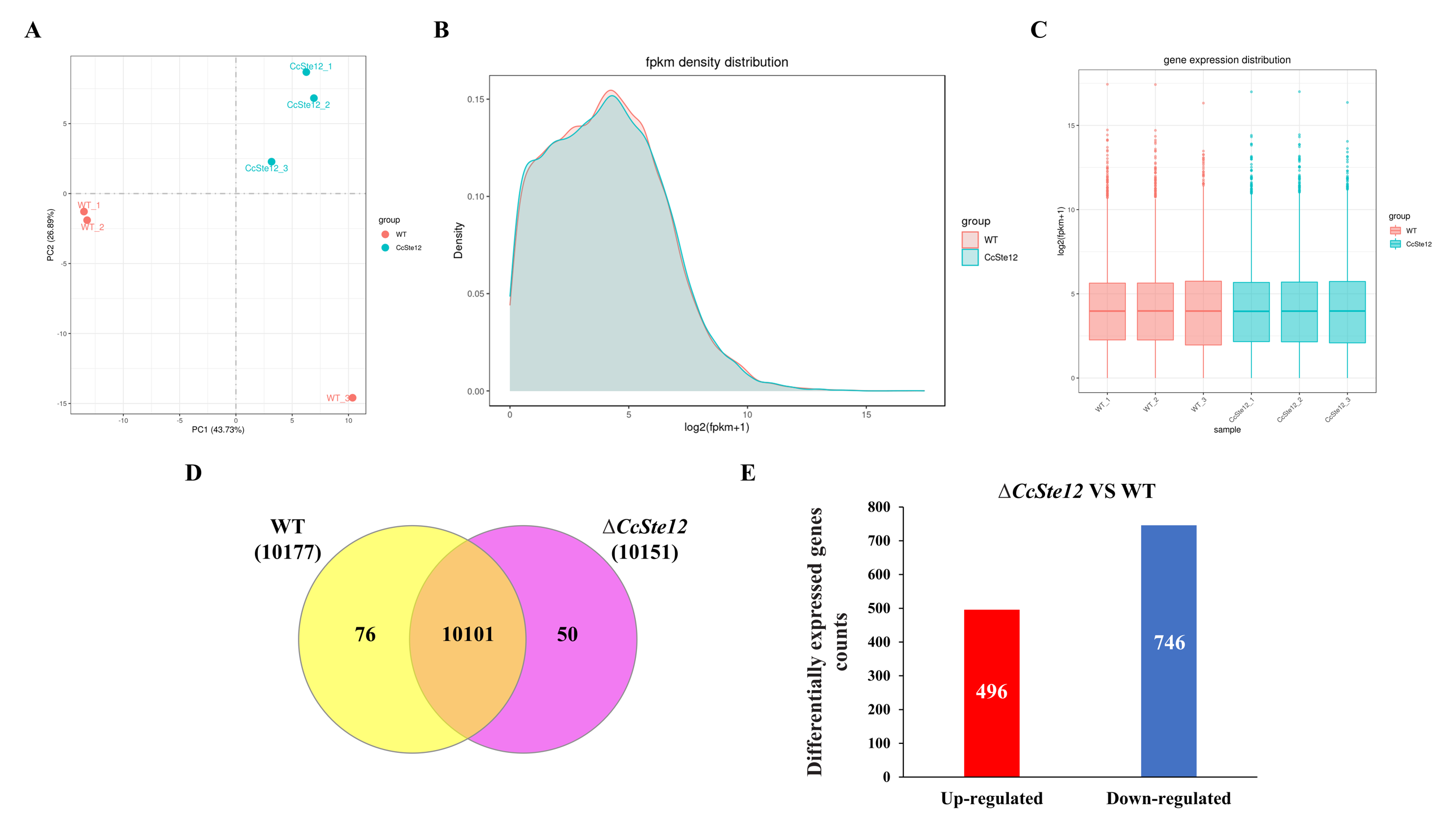

Supplement: Supplementary file 6 — Additional file 6: Figure S6. Gene expression data among wild type and ΔCcSte12. (A) Principal component analysis of the wild-type and ΔCcSte12. (B) The global view of the distribution of gene expression density in the wild-type and ΔCcSte12. (C) The global view of the distribution of gene expression level in the wild-type and ΔCcSte12. (D) The global view of the number of expressed genes in ΔCcSte12 and wild-type. (E) The global view of the number of differential expression genes of ΔCcSte12 and wild-type. [file 44154_2023_142_MOESM6_ESM.tif]

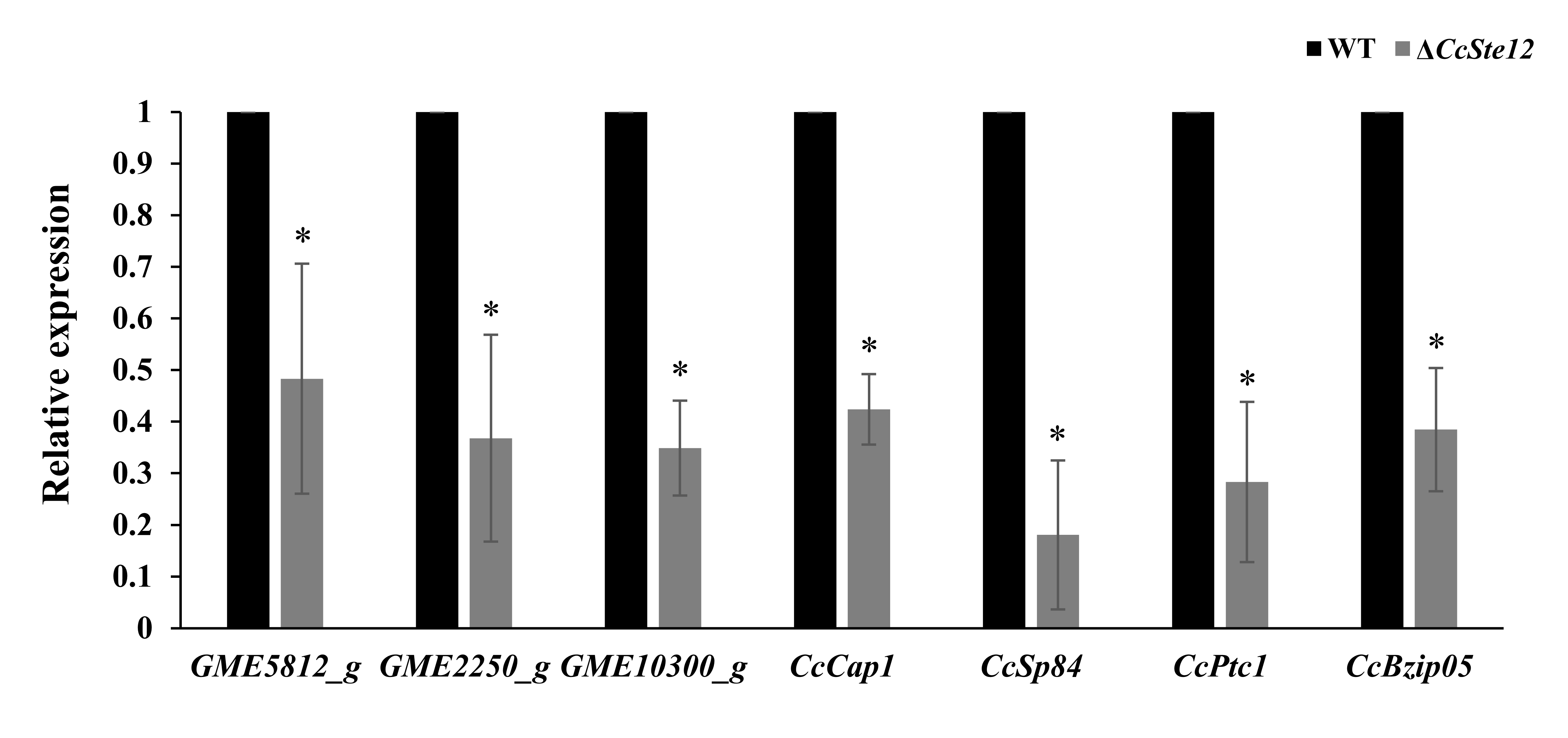

Supplement: Supplementary file 7 — Additional file 7: Figure S7. The RT-qPCR confirmation of RNA-Seq results. The RT-qPCR analysis was conducted and the expression levels of three glycosyl hydrolases (GME5812_g, GME2250_g and GME10300_g), two effectors (CcCap1 and CcSp84) and two genes associated with virulence (CcPtc1 and CcBzip05) were significantly down-regulated in the CcSte12 deletion mutants compared with the wild type. [file 44154_2023_142_MOESM7_ESM.tif]
